# Perceived Ratings of AI-Generated and Expert-Reviewed vs Clinician-Developed Virtual Cardiology Patient Scenarios Among Medical Students: Randomized Controlled Study

**DOI:** 10.2196/100839

**Published:** 2026-10-28

**Authors:** Marios Georgios Bantidos, Eleni Dafli, Efstratios Karagiannidis, Barbara Fyntanidou, Sotiria Gilou, Athina Nasoufidou, Panagiotis Bamidis

**Affiliations:** 1 Lab of Medical Physics and Digital Innovation School of Medicine Aristotle University of Thessaloniki Thessaloniki, Central Macedonia Greece; 2 Department of Emergency Medicine, AHEPA University General Hospital School of Medicine Aristotle University of Thessaloniki Thessaloniki, Central Macedonia Greece; 3 Second Department of Cardiology, Hippokration General Hospital School of Medicine Aristotle University of Thessaloniki Thessaloniki, Central Macedonia Greece

**Keywords:** cardiology, ChatGPT, generative artificial intelligence, generative AI, medical education, randomized controlled trial, RCT, virtual patients, VPs

## Abstract

**Background:**

Virtual patients (VPs) are widely used in medical education, but creating high-quality cases requires clinical expertise, iterative revision, and alignment with educational objectives. Generative AI may support VP authoring, but direct comparisons between AI-generated and clinician-developed cases delivered within the same educational setting remain limited.

**Objective:**

This study aimed to compare medical students’ learner-perceived ratings of 1 AI-generated, expert-reviewed virtual cardiology patient scenario with those of 1 clinician-developed virtual cardiology patient scenario delivered through the same mobile platform.

**Methods:**

A randomized, participant-blinded 2-arm controlled study was conducted in October 2025 during scheduled fifth-year emergency medicine course sessions. Eligible participants were fifth-year medical students who had completed the cardiology course. Students were assigned 1:1 to complete either a clinician-developed VP (CD-VP; n=64) or a ChatGPT-generated VP (GPT-VP; n=67). Both scenarios followed a shared acute coronary syndrome blueprint and were delivered through the same mobile-optimized app (mobile VPs app [MobiViP]) with identical interface structure and branching logic. The GPT-VP was generated using GPT-5 and refined through iterative follow-up prompting under expert review; direct manual rewriting was prohibited. After completion, students completed a mandatory 19-item questionnaire covering realism and validity, cognitive load, self-reported learning and reflection, and engagement and satisfaction. Analyses included reliability testing, 1-sample tests against a neutral midpoint, Mann-Whitney *U* tests, and Spearman correlations. Full scenario content and development details are provided as multimedia appendices.

**Results:**

A total of 131 complete questionnaires were analyzed, with no item-level missing data. Mean subscale scores in the CD-VP group were 3.81 (SD 0.64) for realism and validity, 3.67 (SD 0.74) for cognitive load, 3.64 (SD 0.73) for self-reported learning and reflection, and 4.16 (SD 0.64) for engagement and satisfaction; corresponding GPT-VP scores were 4.11 (SD 0.56), 3.03 (SD 0.76), 3.86 (SD 0.77), and 4.28 (SD 0.58), respectively. Compared with the CD-VP, the GPT-VP received higher ratings for realism and validity (Mann-Whitney *U*=1503.0; *P*=.003) and lower cognitive load ratings, indicating a lower perceived burden (Mann-Whitney *U*=1070.5; *P*<.001). Self-reported learning and reflection showed a marginal difference between groups, with a higher rating for the GPT-VP (Mann-Whitney *U*=1719.0; *P*=.05), whereas engagement and satisfaction did not differ significantly (Mann-Whitney *U*=1922.5; *P*=.31). Internal consistency was acceptable to good for most subscales, although internal consistency for the realism and validity subscale in the GPT-VP group was moderate (Cronbach α=0.67).

**Conclusions:**

In this single-center randomized study, 1 AI-generated, expert-reviewed cardiology VP scenario was rated similarly to or more favorably than 1 clinician-developed scenario in selected learner-perceived domains. Future research should expand to objective learning, retention, development workload, and scalability outcomes.

## Introduction

### Case-Based Learning

Case-based learning (CBL) is a core strategy in health professions education, linking biomedical knowledge with clinical reasoning in authentic contexts. Well-designed cases support diagnostic thinking, guideline-based management, and productive uncertainty, and CBL has been associated with improved learner engagement and performance compared with more didactic approaches [1,2]. However, high-quality cases require time, clinical expertise, iterative editing, and ongoing updates as evidence evolves—factors that often slow content refresh cycles [3].

### Virtual Patients

Virtual patients (VPs), defined as “interactive computer simulations of real-life clinical scenarios for the purpose of health care and medical training, education or assessment,” help scale experiential learning through branching pathways, immediate feedback, and multimedia use [4]. Meta-analyses suggest that VPs are at least noninferior to traditional methods in terms of knowledge outcomes and can strengthen clinical reasoning skills [5,6]. Recent work has also described practical implementation models for undergraduate programs [7]. Still, authoring coherent and realistic VP scenarios remains resource-intensive.

### The Role of Generative AI

Generative AI, particularly large language models (LLMs), such as OpenAI’s GPT models, including ChatGPT, may support educational content authoring by rapidly generating structured text, adapting material to the learner level, and responding to iterative prompting. The use of generative AI in medical education has expanded rapidly, alongside ongoing debate regarding reliability, transparency, and appropriate oversight [8,9]. Documented limitations, including hallucinations, fabricated references, and hypersensitivity to prompting, raise concerns about independent use in training contexts [10,11]. At the same time, improved performance on licensing-style evaluations (eg, GPT models on United States Medical Licensing Examination [USMLE]–aligned tasks) highlights both rapid progress and substantial variability across domains and item types [12-14]. For interactive clinical scenarios, these limitations are especially relevant because errors may affect not only isolated facts but also the sequence, prioritization, and consequences of clinical decisions. Comparative research on AI-generated educational materials remains mixed. Controlled studies of AI-generated multiple-choice questions (MCQs) and vignettes frequently recommend expert review and report variability in difficulty and factual accuracy [15]. Direct comparisons within the same medical domain and delivery platform remain limited [16].

### Evaluation Domains

Evaluation of AI-generated VPs should prioritize the fidelity of the evolving clinical trajectory, guideline concordance, and the plausibility of branching pathways, including consequence-specific corrective (“rescue”) routes. These requirements extend beyond factual recall and place substantial demands on automated scenario generation. Such demands are most apparent in acute care specialties, where decisions are sequential and errors compound rapidly. Cardiology provides a stringent test environment, requiring synchronization of multimodal data with time-critical decisions.

### Study Objective

This study compared medical students’ learner-perceived ratings of 1 AI-generated, expert-reviewed mobile cardiology VP scenario with those of 1 clinician-developed mobile cardiology VP scenario. The aim was to evaluate the acceptability of AI-assisted VP development within a controlled medical education setting.

## Methods

### Study Design and Setting

This 2-arm comparative study evaluated medical students’ assessments of 2 cardiology VP scenarios: a clinician-developed VP (CD-VP) and a ChatGPT-generated VP (GPT-VP). The study was conducted in October 2025 during the fifth-year (undergraduate) emergency medicine course (fall semester). Participants were fifth-year medical students who had completed the cardiology course in the previous academic year. Students accessed the study on their mobile phones via a link distributed during the scheduled teaching session. Scenario allocation was performed using simple 1:1 randomization, with assignment generated at the time of access using a random number generator (randomizer app). Scenarios were presented without authorship labeling, and students were not informed that 1 scenario was AI generated. After providing written informed consent, students were instructed to complete the assigned VP scenario individually and then complete the postscenario questionnaire. No individualized tutoring or content-specific feedback from faculty was provided, and group work was not allowed. Statistical analyses were performed by blinded independent statisticians, and the principal investigators did not have direct access to group-labeled results during analysis. The study is reported in accordance with the 2025 CONSORT (Consolidated Standards of Reporting Trials) statement [17]. Details are provided in Supplementary Appendix S1 in Multimedia Appendix 1. Because both interventions were web based, a completed CONSORT-EHEALTH (Consolidated Standards of Reporting Trials of Electronic and Mobile Health Applications and Online Telehealth) checklist is provided as Multimedia Appendix 2 [18].

### Ethical Considerations

The study was conducted under the overall approval of the Bioethics and Deontology Committee of the Aristotle University of Thessaloniki (266491/2022). As this was an educational, nonclinical, minimal risk study conducted within the scope of the approved research program, a separate ethics approval was not required under institutional procedures. However, the study was reviewed under the institutional data protection and General Data Protection Regulation (GDPR) procedures of the Aristotle University of Thessaloniki (322/31-10-2024). The institutional determination confirmed that the study used anonymized postscenario learner-perceived data collected within scheduled undergraduate teaching and did not involve patients, patient-level clinical outcomes, or identifiable health data. All participants provided written informed consent before participation. Participation was voluntary, and participants received no compensation.

The study was not prospectively registered because it evaluated an educational intervention, did not involve patients or patient-level clinical outcomes, and was not a therapeutic, diagnostic, or health service intervention requiring registration under the applicable institutional and trial registration criteria.

### VP Development and Delivery

To ensure structural and pedagogical comparability, both VP scenarios were developed based on a shared blueprint focused on acute coronary syndrome (ACS), covering identification, clinical progression, and management, and emphasizing high-yield clinical reasoning and learning objectives relevant to undergraduate medical training according to the 2023 European Society of Cardiology guidelines for the management of ACS [19]. Table 1 summarizes key design features of the CD-VP and GPT-VP.

The CD-VP was clinician developed by a 5-member team that included 2 senior professors of medicine, 1 in cardiology and 1 in emergency medicine. The same 2 senior clinical experts also reviewed the GPT-VP, providing shared oversight across both development pathways. The GPT-VP underwent 2 substantive expert review rounds before implementation. In the first round, the full scenario was reviewed for clinical sequence, decision nodes, answer options, automated feedback, rationales, and planned visual material. Review was not blinded to AI authorship because it was conducted as formative quality assurance of the AI-assisted scenario before student use rather than as an independent comparative rating exercise. Expert comments were reconciled through discussion, and final readiness was determined by consensus between the cardiology and emergency medicine experts after the second review round.

The GPT-VP was generated in September 2025 using OpenAI’s ChatGPT (Plus subscription) with GPT-5, using both the standard mode and the extended reasoning “Thinking” setting (GPT-5 Thinking) under constrained prompting. The initial draft underwent structured expert review and targeted revision prior to implementation (Figure 1). Importantly, revisions were implemented through iterative follow-up prompting rather than direct manual modification of the scenario content. Review domains, representative modifications, and prompting workflow details are summarized in Supplementary Appendix S2 in Multimedia Appendix 1. The full CD-VP and GPT-VP scenario contents are provided in Multimedia Appendix 3. The complete chronological ChatGPT conversation history was not exported or retained as a verbatim prompt transcript; therefore, exact prompt-by-prompt reproduction is not possible. To improve transparency, Multimedia Appendix 1 reports the available development record, including the reconstructed initial prompt template, main follow-up prompt categories, mode use approach, 2 substantive expert-guided revision rounds, and criteria used to accept, revise, regenerate, or reject content.

Both scenarios were delivered via the mobile VPs app (MobiViP), a web-based platform optimized for mobile devices that supports branching decision pathways, integrated clinical media, and automated option-specific feedback. The app is available online [20]. The platform environment, user interface, and feedback architecture were standardized across study arms to ensure consistent delivery conditions [21].

**Table 1:** Structural, development, and delivery features of the 2 virtual patient scenarios^a^.

Features	CD-VP^b^	GPT-VP^c^
Development pathway	Clinician developed	AI generated using ChatGPT (GPT-5), followed by expert review and iterative prompt-based revision
Development and/or review team	5-member development team: 1 medical student, 1 resident, 1 fellow, 1 cardiology professor, and 1 emergency medicine professor	2 expert reviewers: 1 cardiology professor and 1 emergency medicine professor, both of whom were senior members of the CD-VP development team
Approximate development calendar time	1 weekend day plus 2 additional afternoons, including team coordination and retrieval and selection of existing clinical media	Half a day, including prompt-based drafting, expert review, and final approval before implementation
Delivery platform	MobiViP^d^	MobiViP
Delivery format	Mobile-optimized branching VP^e^ scenario	Mobile-optimized branching VP scenario
Core clinical domain	ACS^f^ and post-MI^g^ management	ACS and post-MI management
Number of decision points	16	16
Educational level	Undergraduate medical students	Undergraduate medical students
Initial presentation	STEMI^h^-focused acute chest pain presentation	STEMI-focused acute chest pain presentation
Main learning emphasis	ACS recognition, urgent reperfusion, catheterization laboratory decision-making and complication management, subsequent HFrEF^i^ treatment, and device-therapy decisions	ACS recognition, antiplatelet and anticoagulant selection, rhythm and complication management, subsequent HFrEF treatment, second ACS management and treatment algorithm shift, and device-therapy decisions
Clinical media	Electrocardiograms, angiography, echocardiograms, and monitor-based clinical material	Scenario images, chest radiography, and monitor-based clinical material
Automated feedback structure	Option-specific	Option-specific with explanatory rationale
Completion time or time on task	No strict time limit within the practical limits of the scheduled teaching session (individual time on task was not prospectively recorded)	No strict time limit within the practical limits of the scheduled teaching session (individual time on task was not prospectively recorded)
Information volume	Not objectively quantified	Not objectively quantified
Full scenario content	Provided in Multimedia Appendix 3	Provided in Multimedia Appendix 3

^a^Case-specific differences in clinical trajectory, information volume, media, wording, and feedback style may have influenced learner ratings. Approximate development time was retrospectively estimated by the author team and was not prospectively time-logged; therefore, these values are descriptive and should not be interpreted as a formal workload or efficiency comparison.

^b^CD-VP: clinician-developed virtual patient.

^c^GPT-VP: ChatGPT-generated virtual patient.

^d^MobiViP: mobile virtual patients app.

^e^VP: virtual patient.

^f^ACS: acute coronary syndrome.

^g^MI: myocardial infarction.

^h^STEMI: ST-elevation myocardial infarction.

^i^HFrEF: heart failure with reduced ejection fraction.

**Figure 1:**
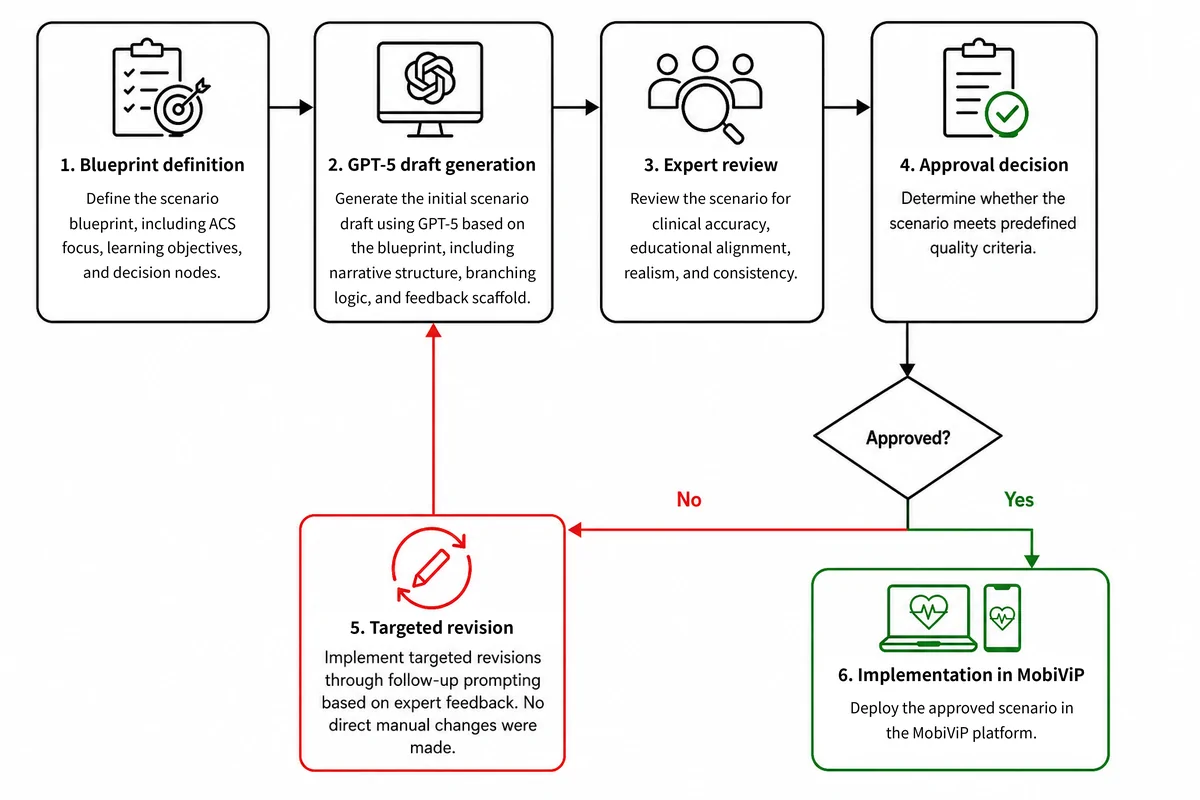
Expert-supervised workflow for development of the ChatGPT-generated virtual patient (GPT-VP). ACS: acute coronary syndrome; MobiViP: mobile virtual patients app.

### Measures and Outcomes

The structured postscenario questionnaire consisted of 5-point Likert scale items (1=“strongly disagree” to 5=“strongly agree”) and was configured with mandatory responses for all items, resulting in no item-level missing data among submitted questionnaires. The full questionnaire is provided in Supplementary Appendix S3 in Multimedia Appendix 1. The instrument comprised 19 items grouped a priori into 4 subscales: (1) realism and validity (3 items), assessing perceived realism, internal coherence, and appropriateness of difficulty; (2) cognitive load (3 items), assessing perceived mental effort, time pressure, and cognitive demands (including sustained attention and recall of clinical knowledge); (3) self-reported learning and reflection (6 items), assessing perceived knowledge gains, identification of knowledge gaps, confidence in clinical decision-making, preparedness for similar cases, and motivation for further study; and (4) engagement and satisfaction (7 items), assessing engagement or immersion, perceived usefulness of feedback, overall satisfaction, and willingness to recommend or further integrate similar VPs into the curriculum. The same questionnaire structure and subscale composition were used for both scenarios to ensure comparability of evaluations. For each participant, subscale scores were calculated as the mean of the corresponding items. The questionnaire was developed for this study to capture learner perceptions rather than to serve as a fully validated measure of objective learning or clinical performance. Item grouping was defined a priori according to the intended constructs, and no claim of full psychometric validation is made.

The primary outcomes were between-scenario differences (CD-VP vs GPT-VP) in the 4 subscale scores. Secondary outcomes included comparisons of subscale scores against the neutral midpoint of the Likert scale (3) and exploratory analyses of within-scenario correlations between subscales.

### Statistical Analysis

Descriptive statistics (sample size, mean values, SDs, and score ranges) were calculated for each subscale by scenario. Normality was assessed using the Shapiro-Wilk test. Internal consistency of each subscale was evaluated using Cronbach alpha, calculated separately for CD-VP and GPT-VP; scores ≥0.70 were considered acceptable.

To examine whether student ratings differed from the neutral midpoint (3), 1-sample tests were performed using Wilcoxon signed-rank tests when normality assumptions were violated; where normality was met (self-reported learning and reflection subscale in the CD-VP), a 1-sample, 2-tailed *t* test was applied. Between-scenario comparisons were performed using Mann-Whitney *U* tests. For interpretability, between-group differences were additionally expressed as a common language effect size (CLES; probability of superiority), derived from the Mann-Whitney *U* statistic and defined as the probability that a randomly selected GPT-VP participant would report a more favorable subscale score than a randomly selected CD-VP participant, with 95% CIs. For cognitive load, “more favorable” was defined as a lower score.

Within-scenario associations between questionnaire subscales were examined using Spearman rank-order correlation coefficients, calculated separately for CD-VP and GPT-VP. The following pairs were examined a priori: realism and validity with self-reported learning and reflection; realism and validity with engagement and satisfaction; self-reported learning and reflection with engagement and satisfaction; cognitive load with self-reported learning and reflection; and cognitive load with engagement and satisfaction.

All tests were 2-tailed with *P*<.05. Because this was an exploratory educational study with prespecified learner-perceived subscales, no formal adjustment for multiple comparisons was applied. Accordingly, statistically significant and marginal findings were interpreted cautiously. Analyses were conducted using SPSS (version 29; IBM Corp) and Python (version 3.13.5; Python Software Foundation).

## Results

All submitted questionnaires were complete and included in the analysis; a total of 64 students completed the questionnaire for the CD-VP and 67 completed the questionnaire for the GPT-VP. Participant flow is shown in Figure 2.

**Figure 2:**
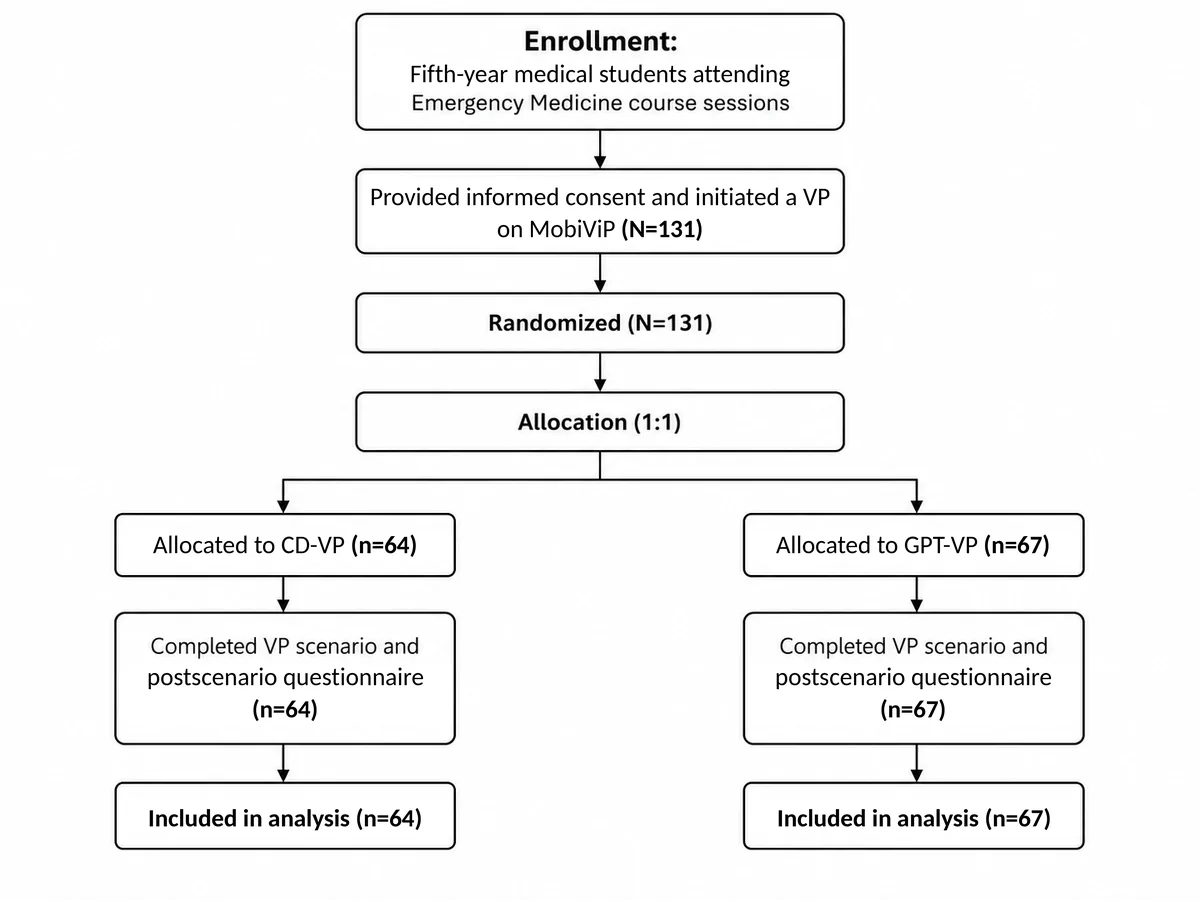
Participant flow diagram. CD-VP: clinician-developed virtual patient; GPT-VP: ChatGPT-generated virtual patient.

### Internal Consistency

Internal consistency for each questionnaire subscale is presented in Table 2. In the CD-VP scenario, Cronbach α indicated acceptable internal consistency for realism and validity (α=0.71) and cognitive load (α=0.70), and good internal consistency for self-reported learning and reflection (α=0.82) and engagement and satisfaction (α=0.87). In the GPT-VP scenario, internal consistency was moderate for realism and validity (α=0.67), acceptable for cognitive load (α=0.70), good for self-reported learning and reflection (α=0.85), and acceptable for engagement and satisfaction (α=0.77).

**Table 2:** Internal consistency (Cronbach α) of questionnaire subscales for the 2 virtual patient scenarios.

Subscales	Clinician-developed virtual patient		ChatGPT-generated virtual patient	
	Cronbach α	Interpretation^a^	Cronbach α	Interpretation^a^
Realism and validity	0.71	Acceptable	0.67	Moderate
Cognitive load	0.70	Acceptable	0.70	Acceptable
Self-reported learning and reflection	0.82	Good	0.85	Good
Engagement and satisfaction	0.87	Good	0.77	Acceptable

^a^Interpretation thresholds: α<0.70 was considered “moderate,” α≥0.70 was considered “acceptable,” and α≥0.80 was considered “good.”

### Descriptive Subscale Scores

Descriptive subscale statistics are presented in Table 3 and visualized in Figure 3. In the CD-VP scenario, mean scores were 3.81 (SD 0.64) for realism and validity, 3.67 (SD 0.74) for cognitive load, 3.64 (SD 0.73) for self-reported learning and reflection, and 4.16 (SD 0.64) for engagement and satisfaction. In the GPT-VP scenario, mean scores were 4.11 (SD 0.56) for realism and validity, 3.03 (SD 0.76) for cognitive load, 3.86 (SD 0.77) for self-reported learning and reflection, and 4.28 (SD 0.58) for engagement and satisfaction. Engagement and satisfaction showed the highest mean score in both scenarios.

**Table 3:** Descriptive statistics of questionnaire subscale scores by virtual patient scenario.

Subscales and scenarios		Score^a^, mean (SD; range)
**Realism and validity**		
	CD-VP^b^	3.81 (0.64; 2.00-5.00)
	GPT-VP^c^	4.11 (0.56; 2.67-5.00)
**Cognitive load**		
	CD-VP	3.67 (0.74; 1.67-5.00)
	GPT-VP	3.03 (0.76; 1.67-5.00)
**Self-reported learning and reflection**		
	CD-VP	3.64 (0.73; 1.50-5.00)
	GPT-VP	3.86 (0.77; 1.67-5.00)
**Engagement and satisfaction**		
	CD-VP	4.16 (0.64; 2.57-5.00)
	GPT-VP	4.28 (0.58; 3.00-5.00)

^a^All questionnaire items were rated on a 5-point Likert scale (1=“strongly disagree” to 5=“strongly agree”).

^b^CD-VP: clinician-developed virtual patient, n=64.

^c^GPT-VP: ChatGPT-generated virtual patient, n=67.

**Figure 3:**
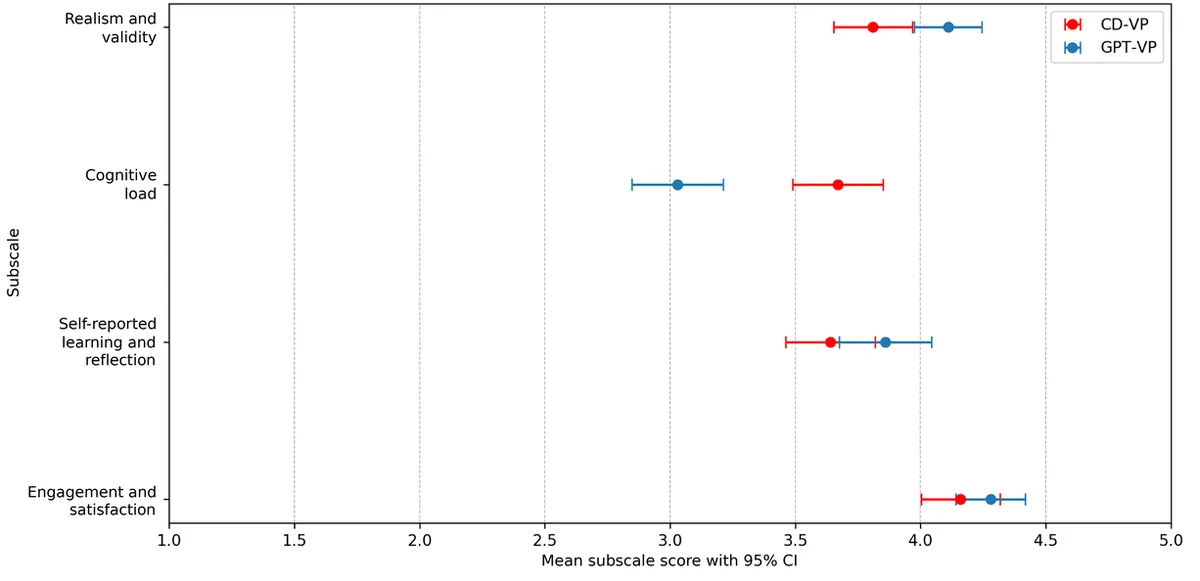
Mean student ratings by subscale for clinician-developed virtual patient (CD-VP) and ChatGPT-generated virtual patient (GPT-VP). Points represent mean subscale scores (on a 5-point Likert scale), and whiskers indicate 95% CIs.

### Comparisons Against the Neutral Midpoint

One-sample analyses examined whether subscale ratings differed from the neutral midpoint of the Likert scale (3). Because normality was violated for all subscales except self-reported learning and reflection in the CD-VP scenario, Wilcoxon signed-rank tests were used for most comparisons, and a 1-sample *t* test was applied where normality assumptions were met.

In the CD-VP scenario, ratings were significantly above the midpoint for realism and validity (*Z*=−6.192; *P*<.001), cognitive load (*Z*=−5.242; *P*<.001), and engagement and satisfaction (*Z*=−6.778; *P*<.001). Self-reported learning and reflection was analyzed using a 1-sample *t* test and was also significantly above the midpoint (t_63_=6.920; *P*<.001), with a mean difference of 0.64 (95% CI 0.45-0.82).

In the GPT-VP scenario, ratings were significantly above the midpoint for realism and validity (*Z*=−6.916; *P*<.001), self-reported learning and reflection (*Z*=−6.052; *P*<.001), and engagement and satisfaction (*Z*=−7.071; *P*<.001). Cognitive load did not differ from the midpoint (*Z*=−0.210; *P*=.83).

### Between-Scenario Comparisons

Between-scenario differences in subscale scores (CD-VP vs GPT-VP) were assessed using Mann-Whitney *U* tests, as the assumption of normality was violated. Realism and validity differed significantly between scenarios (Mann-Whitney *U*=1503.0; *P*=.003), with higher mean ranks in the GPT-VP scenario than in the CD-VP scenario (75.57 vs 55.98). Cognitive load also differed significantly (Mann-Whitney *U*=1070.5; *P*<.001), with higher mean ranks in the CD-VP scenario than in the GPT-VP scenario (82.77 vs 49.98). Self-reported learning and reflection showed a marginal between-scenario difference between groups, with a higher rating for the GPT-VP (Mann-Whitney *U*=1719.0; *P*=.05), with higher mean ranks in the GPT-VP scenario than in the CD-VP scenario (72.34 vs 59.36). Engagement and satisfaction did not differ significantly between scenarios (Mann-Whitney *U*=1922.5; *P*=.31). To aid interpretation, these between-group differences are additionally presented as a CLES (probability of superiority) with 95% CIs (Figure 4).

**Figure 4:**
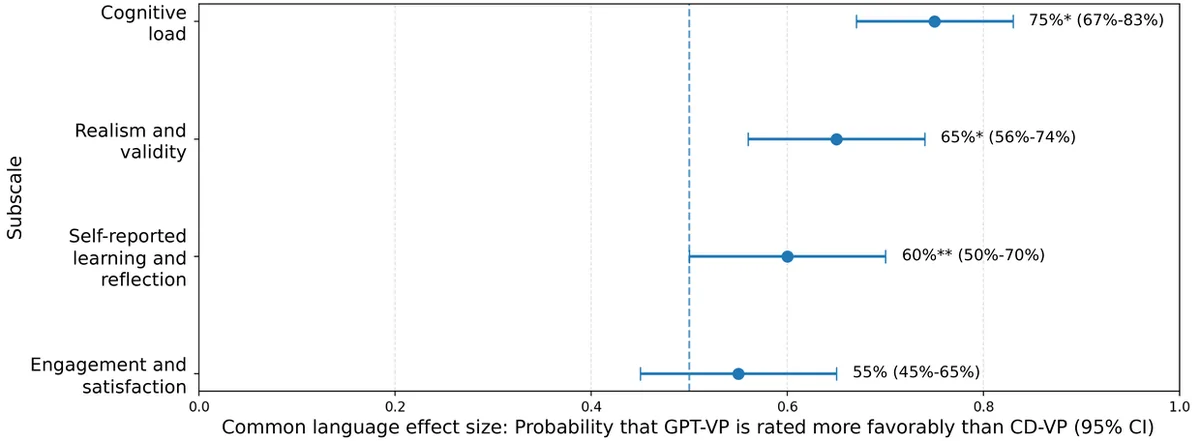
Common language effect size for between-scenario differences (rank-based). Points represent the probability that a randomly selected participant in the ChatGPT-generated virtual patient (GPT-VP) group reports a more favorable rating (subscale score) than a randomly selected participant in the clinician-developed virtual patient (CD-VP) group; whiskers indicate 95% CIs. The vertical reference line at 0.5 indicates no difference between scenarios (equal chance); values >0.5 favor GPT-VP and values <0.5 favor CD-VP. For cognitive load, “more favorable” is defined as a lower score. *P<.05; **P=.05 (marginally significant).

### Within-Scenario Correlations

Within-scenario associations between questionnaire subscales were examined using Spearman rank-order correlation coefficients (ρ; Table 4) and are summarized visually in Figure 5 (CD-VP: lower triangle; GPT-VP: upper triangle). Correlation strength was interpreted using conventional thresholds (ρ≈0.10=“weak,” ρ≈0.30=“moderate,” and ρ≥0.50=“strong”).

In the CD-VP scenario, realism and validity showed strong positive correlations with self-reported learning and reflection (ρ=0.635; *P*<.001) and with engagement and satisfaction (ρ=0.622; *P*<.001). Self-reported learning and reflection was also strongly correlated with engagement and satisfaction (ρ=0.683; *P*<.001). Cognitive load showed a weak negative association with self-reported learning and reflection (ρ=−0.253; *P*=.04) and no significant association with engagement and satisfaction (ρ=−0.094; *P*=.46).

In the GPT-VP scenario, realism and validity correlated moderately with self-reported learning and reflection (ρ=0.387; *P*=.001) and strongly with engagement and satisfaction (ρ=0.510; *P*<.001). Self-reported learning and reflection was strongly correlated with engagement and satisfaction (ρ=0.545; *P*<.001). Cognitive load showed a weak positive association with self-reported learning and reflection (ρ=0.275; *P*=.02) and no significant association with engagement and satisfaction (ρ=0.182; *P*=.14).

**Table 4:** Spearman rank correlations between questionnaire subscales for the clinician-developed virtual patient (CD-VP) and ChatGPT-generated virtual patient (GPT-VP) scenarios.

Subscale pairs	CD-VP, Spearman ρ	*P* value	GPT-VP, Spearman ρ	*P* value
Realism and validity–self-reported learning and reflection	0.635	<.001	0.387	.001
Realism and validity–engagement and satisfaction	0.622	<.001	0.510	<.001
Self-reported learning and reflection–engagement and satisfaction	0.683	<.001	0.545	<.001
Cognitive load–self-reported learning and reflection	−0.253	.04	0.275	.02
Cognitive load–engagement and satisfaction	−0.094	.46	0.182	.14

**Figure 5:**
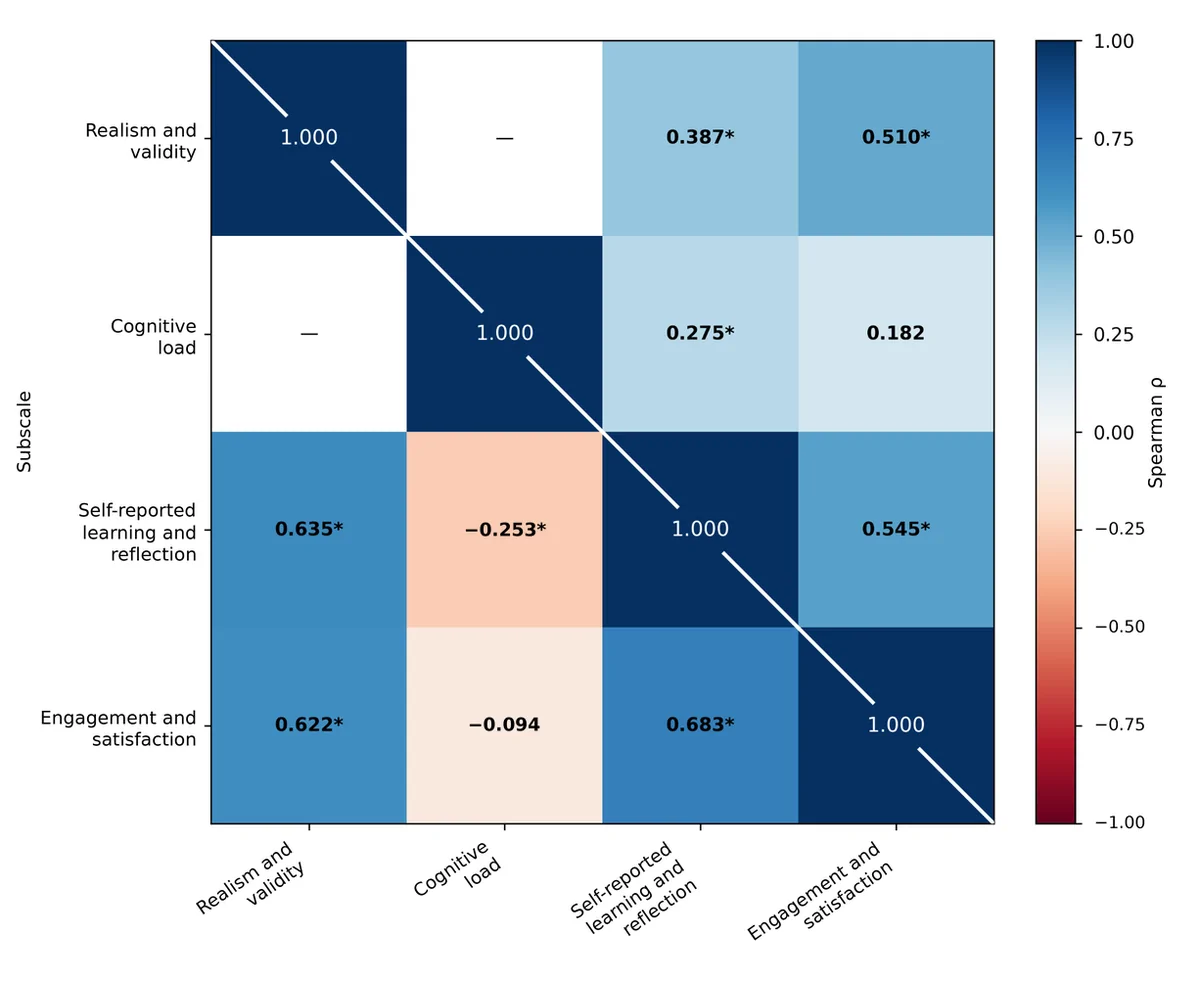
Within-scenario correlations between questionnaire subscales (Spearman ρ). The lower triangle shows correlations for the clinician-developed virtual patient (CD-VP) and the upper triangle shows correlations for the ChatGPT-generated virtual patient (GPT-VP). Cell values are Spearman ρ, and color indicates direction and magnitude of the correlation (−1 to +1). “—” indicates correlations not tested or not reported in Table 4. *P<.05.

## Discussion

### Principal Findings

In this randomized, participant-blinded comparison of 1 CD-VP and 1 expert-reviewed GPT-VP, both scenarios were rated positively by medical students. The questionnaire demonstrated acceptable to good internal consistency, supporting the reliability of the 4 conceptual subscales under these exploratory conditions. Overall, the scenarios were broadly comparable, although the GPT-VP received more favorable ratings in selected domains, including higher realism and validity, lower cognitive load, and marginally higher ratings for self-reported learning and reflection. A graphical abstract summarizing the study is provided in Multimedia Appendix 4.

One plausible interpretation is not that AI-generated content is inherently superior, but that the AI-assisted workflow—standardized prompting followed by expert review—may have produced a more streamlined and internally consistent scenario. This may help explain why the GPT-VP was perceived as more realistic while also imposing less cognitive burden. It is also possible that the GPT-VP benefited from more textbook-aligned phrasing, which may have been more familiar to students and therefore perceived as clearer or more realistic than the more idiomatic clinical language sometimes used by experienced clinicians [22,23]. However, this interpretation should be considered cautiously, as students’ prior clinical exposure may also have influenced these perceptions [24]. By contrast, the similarly high engagement and satisfaction ratings in both scenarios indicate that students found the VP format educationally valuable regardless of authorship.

Comparisons against the neutral midpoint reinforced this overall pattern, with both scenarios rated positively, although cognitive load differed from the midpoint only in the CD-VP, indicating that this case was experienced as more demanding. The within-scenario correlation patterns provide additional insight. In the CD-VP, higher cognitive load was weakly associated with lower perceived learning, whereas in the GPT-VP it showed a weak positive association with learning. This pattern indicates that cognitive demand may have been experienced differently across the 2 cases—more as a burden in the CD-VP and more as a manageable or productive challenge in the GPT-VP.

### Comparison With Existing Literature

The present findings are broadly consistent with prior VP literature, which supports VPs as useful tools for engagement and applied learning in health professions education. In the Digital Health Education Collaboration systematic review and meta-analysis, Kononowicz et al [5] concluded that VPs were at least as effective as traditional education for knowledge outcomes and potentially more effective for skills outcomes, although the overall evidence base remained heterogeneous and of low-to-moderate certainty. Earlier European Commission-funded initiatives, including electronic Virtual Patients (eViP) and mEducator, systematically explored VP development, reuse, sharing, and implementation [25-27]. This work also highlighted the workload associated with authoring and adapting VP scenarios, which can limit adoption by individual clinical teachers and institutions.

In this context, generative AI represents a potentially useful approach. Most empirical work on generative AI in medical education has focused on more static educational outputs than full interactive VPs. In a randomized controlled experiment involving 74 medical students, Coşkun et al [16] found that ChatGPT-generated clinical vignettes did not differ significantly from human-written cases across 10 learner-rated statements, although the associated MCQ set was more variable, with only 6 of 15 items showing acceptable point-biserial discrimination. A similar pattern appears in the assessment literature. In the multinational prospective study by Cheung et al [28], 50 AI-generated and 50 human-authored graduate medical MCQs were rated by 5 independent international assessors. AI-generated questions were produced much faster (20 minutes 25 seconds vs 211 minutes 33 seconds), and overall quality was broadly comparable, although AI items scored significantly lower on relevance (mean 7.56, SD 0.94 vs mean 7.88, SD 0.52; *P*=.04) and showed wider variability. These studies suggest that generative AI can produce educationally usable material, but consistency and educational fit may still depend on review and refinement.

A particularly close comparator to the present work is the recent study by Szydlak et al [29] on ChatGPT-generated vs human-authored concept maps for clinical reasoning training with VPs. Using 10 diverse VP cases, the authors found that ChatGPT-generated maps contained significantly more concepts and connections (*P*<.001), while clinical content quality remained comparable to that of expert-generated maps. However, the AI maps were judged to lack abstraction, prioritization, and contextual alignment, and in some cases were considered to exceed the optimal cognitive load for learners.

The cognitive load pattern observed in the present study is also compatible with previous VP research showing that learner experience depends strongly on how a case is structured and embedded instructionally. In a study of 171 of 183 students, Marei et al [30] found that collaborative VP use after a lecture produced the highest instructional efficiency and the lowest intrinsic and extraneous cognitive load compared with independent VP formats. Fuoad et al [31] similarly reported, in 2 cohorts of 69 and 70 students, that collaborative VP use was associated with higher situational interest and lower extraneous cognitive load than lecture plus independent VP use.

Finally, adjacent simulation research in emergency and cardiovascular education supports the plausibility that well-designed digital cases can produce meaningful gains when evaluated objectively [32]. In a randomized controlled trial of VP simulation in undergraduate emergency medicine, Law et al [33] recruited 138 final-year students and found that the 4-week exposure group improved more than the 2-week group, with a mean score difference of 18 of 100 (95% CI 9.8-26.3). In immersive ACS virtual reality training, Einloft et al [34] studied 247 medical students and found significant gains in knowledge and clinical quality indicators (CQIs), with composite CQI scores improving from 0.47 before training to 0.76 to 0.80 at follow-up.

### Strengths and Limitations

The study has several strengths. It was conducted within routine teaching, enhancing ecological validity [35]. In addition, both scenarios were delivered under the same general conditions and within a shared instructional blueprint, strengthening comparability between study arms. Students were unaware of scenario authorship, including the use of AI in 1 study arm, reducing expectancy bias. The study included a sample size comparable to or exceeding that of most single-center medical education randomized studies with complete response capture across all participants, eliminating missing data concerns.

Several limitations should nonetheless be acknowledged. First, the outcomes were based on student perceptions rather than objective measures of knowledge, clinical reasoning, retention, or performance. Second, although both scenarios shared the same platform, ACS-focused blueprint, delivery conditions, and number of decision points, the study compared only 1 CD-VP and 1 GPT-VP within a single clinical domain and educational context. Observed differences may therefore reflect case-specific features, including clinical difficulty, branching complexity, information volume, wording, feedback style, or visual material, rather than the development pathway alone [36]. This is particularly relevant for cognitive load: lower perceived burden in the GPT-VP may indicate clearer organization and reduced extraneous load, but it may also reflect lower complexity or a more familiar case structure. Third, although the questionnaire showed acceptable to good internal consistency for most subscales, it was developed for this study to capture learner perceptions and was not fully validated as a measure of overall scenario quality. Fourth, the study did not include independent structured educator ratings of final scenario quality and clinical accuracy after implementation. Fifth, the findings reflect expert-supervised AI-assisted VP development rather than unsupervised AI generation, because the GPT-VP was refined through expert review and iterative prompting. Sixth, although approximate development calendar time was retrospectively estimated, the study did not prospectively record person-hours, review and revision burden, prompting duration, or detailed platform analytics. Finally, the full chronological AI prompt history was not exported or retained as a verbatim transcript, limiting exact reproducibility of the development workflow. Future studies should address these limitations through multiscenario comparisons, objective learning outcomes, log-based analytics, and formal evaluation of development workload.

### Future Directions

Future studies should explore whether AI-assisted VP development translates into measurable educational benefits by assessing outcomes such as postintervention test performance, clinical reasoning in novel cases, and longer-term knowledge retention. Future work should also compare the practical efficiency of clinician-only and AI-assisted VP workflows, including revision burden and update cycles.

It will also be important to determine which VP design features are most effective, including branching density, information volume, feedback structure, and media use. Given the potential language barrier, future studies should evaluate whether the present workflow performs similarly across languages, as multilingual medical LLM performance has been shown to vary according to both input language and prompt language [37].

Finally, future research should examine whether, and how quickly, AI-assisted VP authoring can support the incorporation of evolving evidence from big data, digital health, personalized medicine, and precision medicine while maintaining appropriate educational value.

### Conclusions

In this randomized, participant-blinded, controlled study of 2 mobile cardiology VP scenarios, both the CD-VP and GPT-VP cases were rated positively by fifth-year medical students. The AI-generated, expert-reviewed scenario received similarly favorable or more favorable learner-perceived ratings in selected domains. These findings provide initial controlled evidence supporting the acceptability of AI-assisted VP development within routine undergraduate medical education. Future research should investigate objective learning outcomes, longer-term retention measures, development workload data, and platform-based analytics.

## Appendix

Multimedia Appendix 1 Supplementary reporting material, AI prompting and expert revision workflow, and postscenario questionnaire.

Multimedia Appendix 2 CONSORT-eHEALTH checklist (V 1.6.1).

Multimedia Appendix 3 Full virtual patient scenario materials, including the full clinician-developed and AI-generated and expert-reviewed scenarios.

Multimedia Appendix 4 Graphical abstract.

## Abbreviations

ACS: acute coronary syndrome
CBL: case-based learning
CD-VP: clinician-developed virtual patient
CLES: common language effect size
CONSORT: Consolidated Standards of Reporting Trials
CONSORT-EHEALTH: Consolidated Standards of Reporting Trials of Electronic and Mobile Health Applications and Online Telehealth
CQI: clinical quality indicator
eViP: electronic Virtual Patients
GDPR: General Data Protection Regulation
GPT-VP: ChatGPT-generated virtual patient
LLM: large language model
MCQ: multiple-choice question
MI: myocardial infarction
MobiViP: mobile virtual patients app
USMLE: United States Medical Licensing Examination
VP: virtual patient

## Data Availability

Deidentified data may be made available by the corresponding author on reasonable request, subject to institutional and ethical requirements.
