# Supplementary material for: Perceived Ratings of AI-Generated and Expert-Reviewed vs Clinician-Developed Virtual Cardiology Patient Scenarios Among Medical Students: Randomized Controlled Study
Source: JMIR Med Educ. 2026 Oct 28;12:e100839. doi: 10.2196/100839 (PMC13755779; doi:10.2196/100839)
Supplement: Supplementary file 1 [file mededu_v12i1e100839_app2.pdf]

CONSORT-EHEALTH Checklist (V 1.6.1) - Completed Reporting Summary

Manuscript: AI-Generated and Expert-Reviewed Versus Clinician-Developed Mobile Virtual Cardiology Patient Scenarios: Randomized Controlled Study of Medical Student Perceptions

Note: This completed checklist maps CONSORT-EHEALTH V 1.6.1 items to the revised manuscript and multimedia appendices prepared for JMIR Medical Education.

Summary of revised submission package: Main manuscript; Figure 1-5 PNG files; homepage feature image/graphical abstract PNG; Multimedia Appendix 1 containing the CONSORT 2025 reporting summary, AI prompting and expert-revision workflow, and post-scenario questionnaire; Multimedia Appendix 2 containing the full clinician-developed and AI-generated, expert-reviewed VP scenarios; and Multimedia Appendix 3 containing this completed CONSORT-EHEALTH checklist.

Item	CONSORT-EHEALTH reporting area	Location in manuscript/supplement	Manuscript-specific response	Status
**1a**	Title and abstract - identification as randomized trial and mode of delivery	Title; Abstract; Methods 2.2	The revised title identifies the randomized controlled study, the mode of delivery (mobile VP scenarios), the AI-generated and expert-reviewed intervention, the clinician-developed comparator, and the target outcome group (medical student perceptions). The abstract and Methods describe the mobile-optimized MobiViP application and the common platform/interface/branching logic used for both arms.	**Reported**
**1b-i**	Abstract - key features/components of intervention and comparator	Abstract; Methods 2.2; Table 1	The abstract states that students completed either one clinician-developed VP or one AI-generated, expert-reviewed VP. Both followed the same ACS-focused blueprint and were delivered through the same mobile-optimized MobiViP application with identical interface structure and branching logic. Table 1 summarizes key structural, development, and delivery features of both scenarios.	**Reported**
**1b-ii**	Abstract - level of human involvement	Abstract; Methods 2.2; Multimedia Appendix 1, Supplementary Appendix S2	The abstract and Methods state that the GPT-VP was generated using ChatGPT-5 and refined through iterative follow-up prompting under expert review, with direct manual rewriting prohibited. The revised Methods and Multimedia Appendix 1 also report that the complete chronological prompt transcript was not retained, and provide the available development record: reconstructed initial prompt template, representative follow-up prompt categories, standard GPT-5/GPT-5 Thinking mode-use approach, two substantive expert-guided revision rounds, expert-review procedure, intervention-level definitions, and decision criteria.	**Reported**
**1b-iii**	Abstract - recruitment, web-based vs face-to-face components, self-assessed outcomes	Abstract; Methods 2.1-2.3	Participants were fifth-year medical students during scheduled Emergency Medicine course sessions. They accessed the assigned scenario on their own mobile phones and completed a mandatory post-scenario questionnaire immediately after the VP scenario.	**Reported**
**1b-iv**	Abstract - use/uptake data	Abstract; Results	The abstract reports group sizes (CD-VP n=64, GPT-VP n=67) and complete post-scenario questionnaire data with no item-level missing data.	**Reported**
**1b-v**	Abstract - negative trials and uptake	Abstract	Not applicable; this was not a negative trial and both arms completed the assigned VP scenario and questionnaire.	**N/A**
**2a**	Introduction - scientific background, rationale, problem and system type	Introduction 1.1-1.4	The Introduction describes CBL, VPs, the workload involved in VP development, generative AI in medical education, the need for expert oversight, and the rationale for comparing AI-generated and clinician-developed VP scenarios in cardiology.	**Reported**
**2b**	Introduction - specific objectives/hypotheses	Introduction 1.5	The objective is explicitly stated: to compare medical students' learner-perceived ratings of one AI-generated, expert-reviewed VP scenario and one clinician-developed VP scenario delivered through the same mobile platform, providing initial controlled evidence for future multi-scenario and objective-outcome studies.	**Reported**
**3a**	Methods - trial design and allocation ratio	Methods 2.1	Two-arm comparative study with simple 1:1 randomization.	**Reported**
**3b**	Methods - changes after trial commencement; bug fixes/downtime/content changes	Methods 2.1; Results	No technical access problems, platform failures, intervention-delivery failures, or content changes during participant delivery are reported.	**Reported/partly**
**4a-i**	Participants - computer/Internet literacy	Methods 2.1	Participants used their own mobile phones during scheduled sessions. Computer/Internet literacy was not separately assessed.	**Partly reported**
**4a-ii**	Participants - recruitment method; open/closed; web-based vs face-to-face	Methods 2.1	Closed educational setting during scheduled fifth-year Emergency Medicine sessions; students accessed the assigned VP through a study link on their mobile phones.	**Reported**
**4a-iii**	Participants - information given during recruitment and consent	Methods 2.1; Multimedia Appendix 1, Supplementary Appendix S1	Students provided written informed consent before accessing the assigned VP scenario and questionnaire. The study was embedded in routine teaching.	**Reported**
**4b-i**	Settings and locations; online/self-assessed outcomes	Methods 2.1-2.3	Setting was scheduled fifth-year Emergency Medicine course sessions. Outcomes were self-reported using the mandatory post-scenario questionnaire.	**Reported**
**4b-ii**	Institutional affiliations displayed to participants	Methods 2.1	The study was conducted in a university course context. Display of institutional affiliations on the platform was not analyzed as a bias source.	**Partly/N/A**
**5-i**	Interventions - developers, sponsors, owners; relation to study team	Author affiliations; Methods 2.2; References 20-21; COI	MobiViP is cited as a platform of the Lab of Medical Physics and Digital Innovation, Aristotle University of Thessaloniki. Several authors are affiliated with this lab. No conflicts of interest are declared.	**Reported**
**5-ii**	Intervention history/development/formative evaluation	Methods 2.2; References 7 and 21; Multimedia Appendix 1, Supplementary Appendix S2	Prior MobiViP/VP implementation work is cited, and the revised manuscript describes the present CD-VP and GPT-VP development pathways. The GPT-VP development record now includes the prompting approach, expert-guided revision rounds, expert-review procedure, and decision criteria, while explicitly stating that the full chronological prompt transcript was not retained.	**Reported/partly**
**5-iii**	Version/date and content changes/frozen intervention	Methods 2.2; Results	The GPT-VP was generated in September 2025 and deployed for the October 2025 study. No technical access problems or intervention-delivery failures were recorded. A formal software version number is not reported.	**Partly reported**
**5-iv**	Quality assurance methods	Methods 2.2; Table 1; Multimedia Appendix 1, Supplementary Appendix S2	The GPT-VP underwent expert-supervised quality assurance before implementation. The revised manuscript and Appendix S2 report two substantive expert-review rounds, review of the complete scenario, non-blinded formative validation, consensus-based resolution of discrepant comments, definitions of low/moderate/high expert-intervention levels, and final acceptance criteria. Expert review addressed clinical reasoning, content alignment, realism, phrasing, guideline concordance, internal consistency, and feedback quality.	**Reported**
**5-v**	Replicability: screenshots/flowcharts/algorithms/source code	Figures 1-2; Table 1; Multimedia Appendices 1-2	The manuscript provides the GPT-VP workflow figure, participant-flow figure, scenario-comparison table, AI prompting and expert-revision workflow, post-scenario questionnaire, and full CD-VP and GPT-VP scenario content. Exact prompt-by-prompt reproduction is limited because the complete chronological prompt transcript was not retained. Source code is not provided.	**Reported/partly**

Item	CONSORT-EHEALTH reporting area	Location in manuscript/supplement	Manuscript-specific response	Status
**5-vi**	Digital preservation/URL/application access	Methods 2.2; Reference 20	The app URL is provided in the manuscript reference list. Archival/demo access is not reported.	**Partly reported**
**5-vii**	Access to application and setting/context	Methods 2.1	Students accessed the assigned VP scenario on their own mobile phones using a study link distributed during scheduled teaching sessions. No payment was involved.	**Reported**
**5-viii**	Mode of delivery, features, components, tailoring/feedback	Abstract; Methods 2.2	MobiViP is described as a web-based, mobile-optimized platform supporting branching decision pathways, integrated clinical media, and automated option-specific feedback.	**Reported**
**5-ix**	Use parameters/dose/timing	Methods 2.1-2.3	Participants completed one assigned VP scenario during the scheduled course session and immediately completed the post-scenario questionnaire.	**Reported**
**5-x**	Human involvement/support/co-intervention	Methods 2.2; Table 1; Multimedia Appendix 1, Supplementary Appendix S2	Expert involvement occurred during GPT-VP development and review before implementation. Students used the platform under standardized session conditions. No individualized tutoring/support was provided during scenario completion beyond the automated option-specific feedback embedded in the platform.	**Reported**
**5-xi**	Prompts/reminders	Methods 2.1	No ongoing prompts or reminders were used; access was through a study link during the scheduled session.	**N/A/reported**
**5-xii**	Co-interventions, training/support	Methods 2.1	Both groups used the same MobiViP platform environment and assigned scenario structure under the same course-session conditions.	**Reported**
**6a-i**	Outcomes - questionnaire and online deployment/validation	Methods 2.3; Multimedia Appendix 1, Supplementary Appendix S3	The 19-item mandatory post-scenario questionnaire and its four prespecified subscales are described; item wording is provided in Multimedia Appendix 1, Supplementary Appendix S3. The revised manuscript states that the questionnaire captured learner perceptions and was not a fully validated measure of objective learning or overall VP quality.	**Reported/partly**
**6a-ii**	Outcomes - use/intensity metrics	Methods 2.3; Results	All analyzed participants completed the assigned scenario and questionnaire. No detailed log-file analytics such as time on platform or clickstream data were collected.	**Reported/limited**
**6a-iii**	Outcomes - qualitative feedback	Methods/Results	No qualitative feedback collection is reported.	**N/A/not collected**
**6b**	Changes to outcomes after commencement	Methods 2.3; Multimedia Appendix 1, Supplementary Appendix S1	Primary and secondary outcomes were prespecified as between-scenario subscale differences and related analyses; no outcome changes are reported.	**Reported**
**7a**	Sample size determination/expected attrition	Methods/Results; Multimedia Appendix 1, Supplementary Appendix S1	The study included all complete submitted questionnaires from 131 students. A formal a priori sample-size calculation is not reported. Attrition was not observed among submitted questionnaires.	**Partly reported**
**7b**	Interim analyses/stopping rules	Multimedia Appendix 1, Supplementary Appendix S1	No interim analyses or stopping rules were applied because the study was a minimal-risk educational intervention conducted during routine teaching.	**Reported**
**8a**	Random sequence generation	Methods 2.1; Multimedia Appendix 1, Supplementary Appendix S1	Simple randomization was generated at the time of scenario access using a random number generator application.	**Reported**
**8b**	Type of randomization/restrictions	Methods 2.1	Simple 1:1 randomization; no blocking or restriction reported.	**Reported**
**9**	Allocation concealment mechanism	Methods 2.1	Assignment was generated at the time of access using a random number generator application. Further allocation concealment procedures are not described.	**Partly reported**
**10**	Implementation: sequence generation, enrollment, assignment	Methods 2.1	Students accessed the study link during the session and assignment was generated at the time of access. Specific personnel roles for enrollment/assignment are not detailed.	**Partly reported**
**11a-i**	Blinding	Methods 2.1; Multimedia Appendix 1, Supplementary Appendix S1	Participants were blinded to scenario authorship and did not know that one scenario was AI-generated. Statistical analyses were performed by independent statisticians blinded to group labeling during analysis.	**Reported**
**11a-ii**	Blinding and informed-consent expectations	Methods 2.1	Scenarios were presented without authorship labeling, and students were not informed that one scenario was AI-generated.	**Reported**
**11b**	Similarity of interventions	Methods 2.2; Table 1	Both scenarios used the same ACS-focused blueprint, MobiViP platform environment, user interface, feedback architecture, branching VP format, and 16 decision points. Table 1 summarizes key similarities and differences between scenarios.	**Reported**
**12a**	Statistical methods/missing data/imputation	Methods 2.4; Results	Analyses used reliability testing, one-sample tests, Mann-Whitney U tests, CLES with 95% CIs, and Spearman correlations. No imputation was required because submitted questionnaires were complete with no item-level missing data. The revised analysis notes that no formal adjustment for multiple comparisons was applied and findings were interpreted cautiously.	**Reported**
**12b**	Additional analyses	Methods 2.4	Exploratory within-scenario Spearman correlation analyses were prespecified for selected subscale pairs.	**Reported**
**X26**	Ethics and informed consent	Methods 2.1; Multimedia Appendix 1, Supplementary Appendix S1	The study was conducted under the overall approval of the Bioethics and Deontology Committee of the Aristotle University of Thessaloniki (Approval No. 266491/2022). As this was an educational, non-clinical, minimal-risk study conducted within the scope of the approved research program, a separate ethics approval was not required under institutional procedures. The study was also reviewed under the institutional data-protection/GDPR procedures of the Aristotle University of Thessaloniki, with reference number 322/31-10-2024. Students provided written informed consent before accessing the assigned VP scenario and questionnaire.	**Reported**
**13a**	Participant flow	Results; Figure 2	Participant flow reports 131 randomized/initiated/completed/included students, with CD-VP n=64 and GPT-VP n=67.	**Reported**
**13b**	Losses/exclusions after randomization	Results; Figure 2; Multimedia Appendix 1, Supplementary Appendix S1	All submitted questionnaires were complete and included; no item-level missing data. No post-randomization losses among analyzed participants are reported.	**Reported**
**14a**	Recruitment and follow-up dates/secular events	Methods 2.1	The study was conducted in October 2025 during scheduled fifth-year Emergency Medicine sessions. No relevant secular events are reported.	**Reported**
**14b**	Why trial ended/stopped	Methods 2.1	The study was conducted during predefined scheduled course sessions; no early stopping occurred.	**Reported/N/A**

Item	CONSORT-EHEALTH reporting area	Location in manuscript/supplement	Manuscript-specific response	Status
**15**	Baseline demographic/digital divide data	Methods/Results	Eligibility and academic year are reported. No detailed demographic or digital-literacy baseline table is included.	**Partly/not reported**
**16**	Numbers analyzed and denominators/use thresholds	Results; Figure 2	All 131 complete questionnaires were analyzed by assigned scenario: CD-VP n=64 and GPT-VP n=67. Use was operationalized as completion of the assigned VP scenario and post-scenario questionnaire.	**Reported**
**17a**	Outcomes and estimation, including process outcomes	Results 3.1-3.5; Tables 2-4; Figures 3-5	Subscale scores, reliability, between-scenario comparisons, CLES with 95% CIs, and correlations are reported. Process outcomes are limited to scenario/questionnaire completion and absence of item-level missing data.	**Reported/limited**
**17b**	Binary outcomes	Results	Not applicable; primary and secondary outcomes were continuous/rank-based subscale scores, not binary outcomes.	**N/A**
**18**	Ancillary analyses	Methods 2.4; Results 3.5	Within-scenario Spearman correlation analyses were reported separately for CD-VP and GPT-VP.	**Reported**
**19**	Harms/unintended effects, privacy breaches, technical problems	Methods/Results	No technical access problems, platform failures, or intervention-delivery failures were recorded. No harms or privacy breaches were reported.	**Reported**
**20**	Limitations	Discussion 4.3	Limitations include perception-based outcomes, comparison of only one scenario per development pathway, absence of objective learning or retention outcomes, possible case-specific differences in complexity and feedback style, questionnaire validation limits, lack of independent structured educator ratings after implementation, lack of prospective person-hour/revision-burden/platform-analytics data, expert-reviewed AI generation rather than unsupervised AI generation, and absence of a complete chronological prompt transcript.	**Reported**
**21**	Generalizability	Discussion 4.3-4.4	The manuscript discusses transferability limits from one clinical domain, one educational context, and one scenario per development pathway, and suggests future multi-scenario, cross-topic, cross-institution comparisons with objective outcomes and platform analytics.	**Reported**
**22**	Interpretation/principal findings	Discussion 4.1	The Discussion summarizes the principal findings and interprets the pattern of realism, cognitive load, learning/reflection, and engagement results as learner-perceived outcomes from one expert-reviewed AI-generated VP compared with one clinician-developed VP.	**Reported**
**23**	Registration	Methods 2.1; Multimedia Appendix 1, Supplementary Appendix S1	The study was not prospectively registered because it evaluated an educational intervention, did not involve patients or patient-level clinical outcomes, and was not a therapeutic, diagnostic, or health-service intervention requiring registration under the applicable institutional and trial-registration criteria.	**Reported**
**24**	Protocol availability	Manuscript; Multimedia Appendices	No separate full trial protocol is reported. The CONSORT 2025 reporting summary is provided in Multimedia Appendix 1, and the full CD-VP and GPT-VP scenario content is provided in Multimedia Appendix 2. Representative AI prompting workflow details, mode-use approach, expert-review procedure, and decision criteria are provided in Multimedia Appendix 1, Supplementary Appendix S2. The completed CONSORT-EHEALTH checklist is provided as Multimedia Appendix 3.	**Not available**
**25**	Funding	Funding section	No external funding was received.	**Reported**
**X27**	Competing interests and relation to evaluated system	Conflicts of Interest; Author affiliations; Methods 2.2	Authors declare no conflict of interest. Authors are affiliated with the institution/lab associated with the MobiViP platform; the platform is cited in the manuscript.	**Reported/partly**

Prepared for upload as a supplementary file in response to the editorial request for the CONSORT-EHEALTH checklist (V 1.6.1).
