# Supplementary material for: Perceived Ratings of AI-Generated and Expert-Reviewed vs Clinician-Developed Virtual Cardiology Patient Scenarios Among Medical Students: Randomized Controlled Study
Source: JMIR Med Educ. 2026 Oct 28;12:e100839. doi: 10.2196/100839 (PMC13755779; doi:10.2196/100839)
Supplement: Supplementary file 2 [file mededu_v12i1e100839_app1.docx]

**Multimedia Appendix 1**

**Supplementary Reporting Material, AI Prompting and Expert-Revision Workflow, and Post-scenario Questionnaire**

*Manuscript: AI-Generated and Expert-Reviewed Versus Clinician-Developed Mobile Virtual Cardiology Patient Scenarios: Randomized Controlled Study of Medical Student Perceptions*

## Contents

Supplementary Appendix S1. CONSORT 2025 Reporting and Study Conduct Summary.

Supplementary Appendix S2. AI Prompting, Expert Review, and Revision Workflow for GPT-VP Development.

Supplementary Appendix S3. Post-scenario Questionnaire.

# Supplementary Appendix S1. CONSORT 2025 Reporting and Study Conduct Summary

| **CONSORT reporting domain** | **Study-specific information** |
| --- | --- |
| Reporting guideline | This study was reported in accordance with the CONSORT 2025 statement for randomized trials. |
| Study design | Two-arm, parallel-group, randomized comparative study. |
| Study setting | The study was conducted during scheduled fifth-year Emergency Medicine course sessions in October 2025. |
| Participants | Eligible participants were fifth-year medical students who had completed the Cardiology course in the previous academic year. |
| Interventions | Participants completed either a CD-VP or a GPT-VP. |
| Allocation ratio | Participants were assigned in a 1:1 ratio. |
| Randomization method | Simple randomization was generated at the time of scenario access using a random number generator application. |
| Blinding | Participants were blinded to scenario authorship, including the use of AI in one study arm. Statistical analyses were performed by independent statisticians blinded to group labeling during analysis. |
| Outcomes | The primary outcomes were between-scenario differences in four questionnaire subscale scores: Realism and Validity, Cognitive Load, Self-reported Learning and Reflection, and Engagement and Satisfaction. |
| Participant flow | A total of 131 students completed the study questionnaire and were included in the analysis: 64 in the CD-VP group and 67 in the GPT-VP group. Participant flow is shown in Figure 2. |
| Attrition and missing data | All submitted questionnaires were complete, with no item-level missing data. |
| Trial registration and ethical approval | The study was conducted under the overall approval of the Bioethics and Deontology Committee of the Aristotle University of Thessaloniki (Approval No. 266491/2022). As this was an educational, non-clinical, minimal-risk study conducted within the scope of the approved research program, a separate ethics approval was not required under institutional procedures. However, the study was reviewed under the institutional data-protection/GDPR procedures of the Aristotle University of Thessaloniki. The institutional determination confirmed that the study used anonymized post-scenario learner-perception data collected within scheduled undergraduate teaching and did not involve patients, patient-level clinical outcomes, or identifiable health data (reference number: 322/31-10-2024). The study was not prospectively registered because it evaluated an educational intervention, did not involve patients or patient-level clinical outcomes, and was not a therapeutic, diagnostic, or health-service intervention requiring registration under the applicable institutional and trial-registration criteria. |
| Interim analyses and stopping rules | No interim analyses or stopping rules were applied because the study was a minimal-risk educational intervention conducted during routine teaching. |
| AI-assisted intervention development | The GPT-VP was generated using ChatGPT-5 under structured prompting and iterative expert-guided refinement. Direct manual rewriting of scenario content was prohibited; revisions were implemented through follow-up prompting based on expert feedback. |
| Expert review domains | Expert review addressed clinical reasoning, content alignment, realism, phrasing, guideline concordance, and internal consistency. |

**Note.** AI = artificial intelligence; CD-VP = clinician-developed virtual patient; CONSORT = Consolidated Standards of Reporting Trials; GPT-VP = ChatGPT-generated virtual patient; VP = virtual patient.

# Supplementary Appendix S2. AI Prompting, Expert Review, and Revision Workflow for GPT-VP Development

## S2.1 Overview

The GPT-VP was developed through a structured AI-assisted workflow using ChatGPT-5 in September 2025. The workflow included initial scenario generation, expert review, targeted prompt-based revision, and final approval before implementation in MobiViP. Direct manual rewriting of scenario content was prohibited; revisions were implemented through follow-up prompts based on expert feedback.

The complete chronological ChatGPT conversation history was not exported or retained as a verbatim prompt transcript during scenario development. Therefore, exact prompt-by-prompt reproduction, including the exact initial user-entered prompt and the exact chronological alternation between standard GPT-5 and GPT-5 Thinking, is not possible. Instead, this appendix reports the available development record: a reconstructed initial prompt template, main follow-up prompt categories, the mode-use approach, two substantive expert-guided revision rounds, expert-guided refinement targets, and the criteria used to accept, revise, regenerate, or reject content.

## S2.2 Development and Revision Cycle

| **Workflow stage** | **Purpose** | **Output** |
| --- | --- | --- |
| Blueprint definition | Define ACS-focused educational scope, learner level, and sequential decision points | Shared instructional blueprint |
| Initial GPT-5 draft generation | Generate the first interactive VP draft with clinical narrative, answer options, and feedback scaffold | Initial GPT-VP draft |
| Expert review | Assess clinical accuracy, educational alignment, realism, guideline concordance, internal consistency, and feedback quality | Expert comments and revision targets |
| Targeted prompt-based revision | Convert expert comments into follow-up prompts without direct manual rewriting | Revised GPT-VP draft |
| Approval decision | Determine whether the revised case met predefined clinical and educational criteria | Approved or returned for further prompting |
| MobiViP implementation | Deploy the approved scenario in the same platform environment as the CD-VP | Final GPT-VP scenario |

## S2.3 Available Prompting Record, Mode Use, Revision Rounds, and Decision Criteria

Available prompting record. The exact initial user-entered prompt was not retained verbatim. The following reconstructed template reflects the retained development record and the intended constraints used for initial generation: “Create an undergraduate-level interactive cardiology virtual patient scenario focused on acute coronary syndrome. The scenario should be suitable for fifth-year medical students and should include a sequential clinical narrative, 16 decision points, plausible answer options, one best answer per decision point, automated option-specific feedback, and guideline-concordant management. Cover ACS recognition, initial STEMI management, rhythm complications, post-myocardial-infarction heart-failure treatment, recurrent ACS, and secondary prevention. Keep the scenario clinically realistic, internally consistent, and appropriate for implementation in a mobile virtual patient platform.”

Mode use. Standard GPT-5 was used for initial scenario drafting and straightforward targeted revisions, such as rephrasing answer options, improving feedback clarity, and adjusting scenario wording. GPT-5 Thinking was used for more complex clinical coherence checks, including sequencing of acute coronary syndrome management, complication logic, guideline concordance, distractor plausibility, and final readiness checking. The exact prompt-by-prompt alternation between standard GPT-5 and GPT-5 Thinking was not retained.

Revision rounds. The GPT-VP underwent two substantive expert-guided revision rounds after the initial AI-generated draft. Round 1 focused on clinical sequence, decision nodes, distractor plausibility, feedback quality, and realism of clinical data. Round 2 focused on whether the revised scenario had resolved the identified issues and was ready for implementation in MobiViP. Minor formatting adjustments for platform entry were performed after approval, without direct manual rewriting of scenario content.

Decision criteria. Content was accepted when it was clinically accurate, guideline-concordant, internally consistent, appropriate for undergraduate medical students, and technically suitable for implementation in MobiViP. Content was revised when the clinical direction was acceptable but wording, sequencing, distractors, feedback, or clinical details required clarification. Content would have been regenerated if a scenario node was structurally unsuitable despite targeted revision. Content would have been rejected if it contained clinically unsafe, contradictory, or educationally inappropriate material that could not be corrected through prompting. No final implemented node required rejection after expert-guided revision.

## S2.4 Representative Prompting Instructions

| **Prompting step** | **Representative instruction** |
| --- | --- |
| Initial scenario generation | “Create an undergraduate-level interactive cardiology virtual patient scenario focused on acute coronary syndrome. Include sequential decision points, plausible answer options, one best answer, automated option-specific feedback, and guideline-concordant management.” |
| Blueprint alignment | “Ensure that the scenario covers ACS recognition, initial STEMI management, rhythm complications, heart-failure treatment after myocardial infarction, recurrent ACS, and secondary prevention.” |
| Decision-node structure | “For each decision point, provide three answer options where possible, with one best answer and clinically plausible distractors. Avoid making incorrect options obviously wrong unless the teaching objective requires recognition of a dangerous error.” |
| Feedback refinement | “For each incorrect option, provide concise corrective feedback that guides the learner toward the correct next step”. |
| Clinical realism | “Revise vital signs, laboratory values, ECG descriptions, and clinical progression so that they remain physiologically plausible and internally consistent across the case.” |
| Guideline concordance | “Check the management recommendations against contemporary European Society of Cardiology guidance for acute coronary syndrome, post-myocardial-infarction care, heart failure with reduced ejection fraction, and device-therapy decision-making.” |
| Expert-guided revision | “Revise the scenario according to expert comments, focusing on clinical sequencing, vital signs, laboratory values, guideline concordance, clarity of wording, and internal consistency.” |
| Final readiness check | “Check the final scenario for contradictions, unclear decision points, inappropriate distractors, missing feedback, mismatch between clinical findings and recommended management, and suitability for undergraduate medical students.” |

## S2.5 Expert-Review Domains and Representative Modifications

| **Area examined** | **Level of expert intervention** | **Representative expert-review focus** |
| --- | --- | --- |
| Clinical reasoning | Moderate | “Tighten edge-case differentials”. |
| Content | Low-moderate | “Align investigations and clinical findings with objectives”. |
| Realism | Low | “Adjust vital-sign dynamics and laboratory values”. |
| Phrasing | Moderate | “Simplify ambiguous wording”. |
| Guideline concordance | Low | “Add concise justifications for key recommendations”. |
| Consistency | Low | “Maintain blueprint sequencing”. |

## S2.6 Expert-Review Procedure and Intervention-Level Definitions

Expert-review procedure. Two substantive expert-review rounds were completed before implementation in MobiViP. In the first round, the cardiology and emergency medicine experts reviewed the complete GPT-VP scenario, including clinical progression, decision nodes, answer options, automated feedback, rationales, and planned visual material. Reviewer comments were translated into targeted follow-up prompts. In the second round, the revised scenario was assessed for clinical accuracy, guideline concordance, educational alignment, internal consistency, feedback quality, and readiness for student use. Review was not blinded to AI authorship because the purpose was formative expert validation of the AI-assisted scenario before implementation. Discrepant comments were discussed between the two experts, and the final approval decision was made by consensus.

Definition of intervention levels. Low intervention referred to minor wording, clarification, or alignment changes that did not alter the clinical pathway. Moderate intervention referred to changes affecting clinical sequencing, distractor plausibility, feedback rationale, physiologic realism, or guideline-concordant decision logic. High intervention would have referred to rejection or full regeneration of a scenario segment because of clinically unsafe, contradictory, or educationally unsuitable content. No high-level intervention was required for the final implemented GPT-VP.

## S2.7 Examples of Scenario Nodes Selected for Expert-Guided Refinement

| **Scenario area** | **Reason for expert-guided refinement** | **Examples of prompting/refinement focus** |
| --- | --- | --- |
| Initial STEMI management | Increase urgency and avoid overly straightforward distractors | “Emphasize that PCI should not be delayed for serial troponins or echocardiography”. |
| P2Y12 selection | Clarify the clinical rationale behind antiplatelet choice | “Make distinctions between ticagrelor, prasugrel, and clopidogrel more explicit”. |
| Inferior STEMI bradycardia | Improve physiologic realism and escalation logic | “Clarify why atropine is first-line before vasopressors or pacing in the stable initial step.” |
| Acute kidney injury after HFrEF therapy | Add clinical nuance and avoid oversimplified management | “Emphasize volume-status assessment and temporary withholding of ACE inhibitor when appropriate”. |
| QTc prolongation | Emphasize reversible causes | “Avoid inappropriate amiodarone or device-based treatment”. |
| CRT-D eligibility | Clarify device decision-making | “Incorporate LVEF, QRS duration, LBBB, and optimized medical therapy”. |
| Recurrent NSTEMI and secondary prevention | Extend the scenario beyond the acute event | “Include anticoagulation choice and LDL-C target after recurrent ACS”. |

**Note.** ACE inhibitor = angiotensin-converting enzyme inhibitor; ACS = acute coronary syndrome; AI = artificial intelligence; ARB = angiotensin receptor blocker; CD-VP = clinician-developed virtual patient; CRT-D = cardiac resynchronization therapy with defibrillator; GPT-VP = ChatGPT-generated virtual patient; HFrEF = heart failure with reduced ejection fraction; LBBB = left bundle branch block; LDL-C = low-density lipoprotein cholesterol; LVEF = left ventricular ejection fraction; MobiViP = mobile virtual patients app; MRA = mineralocorticoid receptor antagonist; NSTEMI = non-ST-elevation myocardial infarction; PCI = percutaneous coronary intervention; STEMI = ST-elevation myocardial infarction; SVT = supraventricular tachycardia; VP = virtual patient.

# Supplementary Appendix S3. Post-scenario Questionnaire

| **Item** | **Statement** |
| --- | --- |
| **Realism and Validity** |  |
| 1 | The scenario was realistic / the clinical presentation resembled a real case. |
| 2 | The scenario had a logical and coherent flow. |
| 3 | The level of difficulty was appropriate for undergraduate medical students. |
| **Cognitive Load** |  |
| 4 | The scenario required sustained concentration. |
| 5 | I felt time pressure while completing the scenario. |
| 6 | I had to process a large volume of clinical information. |
| **Self-reported Learning and Reflection** |  |
| 7 | The scenario increased my clinical knowledge. |
| 8 | The questions highlighted important educational points. |
| 9 | The scenario increased my confidence in making clinical decisions. |
| 10 | I feel better prepared for similar real-life cases. |
| 11 | The scenario helped me identify knowledge gaps. |
| 12 | The scenario motivated me to study the topic further. |
| **Engagement and Satisfaction** |  |
| 13 | I found the scenario interesting. |
| 14 | I was fully immersed during the scenario. |
| 15 | The interactive platform made the learning experience enjoyable. |
| 16 | I was satisfied with the visual material accompanying each question. |
| 17 | The feedback after each question improved my understanding and helped me progress through the scenario. |
| 18 | I would recommend completing this scenario to other students. |
| 19 | I would like similar scenarios to be integrated into the curriculum for training. |
