# Supplementary material for: Perceived Ratings of AI-Generated and Expert-Reviewed vs Clinician-Developed Virtual Cardiology Patient Scenarios Among Medical Students: Randomized Controlled Study
Source: JMIR Med Educ. 2026 Oct 28;12:e100839. doi: 10.2196/100839 (PMC13755779; doi:10.2196/100839)
Supplement: Supplementary file 3 [file mededu_v12i1e100839_app3.docx]

**Multimedia Appendix 3**

**Full Virtual Patient Scenario Materials**

*Manuscript: AI-Generated and Expert-Reviewed Versus Clinician-Developed Mobile Virtual Cardiology Patient Scenarios: Randomized Controlled Study of Medical Student Perceptions*

## Contents

Section A. Full Clinician-Developed Virtual Patient Scenario (CD-VP).

Section B. Full AI-Generated, Expert-Reviewed Virtual Patient Scenario (GPT-VP).

This appendix provides the complete question-by-question content of both virtual patient scenarios, including correct responses, incorrect-response feedback, rationales where applicable, and visual material as implemented in MobiViP.

# Section A. Full Clinician-Developed Virtual Patient Scenario (CD-VP)

| **CD-VP: Question No.** | **Correct response** | **Incorrect Responses & Feedback** |
| --- | --- | --- |
| **1.** You are a Cardiology resident on a general on-call shift in the ER. A 68-year-old man presents in pain, pointing to his chest. He says:  “I started feeling pain 3 days ago but now it’s much worse. It’s tight and I’m struggling to breathe.”  The ECG you requested is shown below. What is your next step?  A) Immediate transfer to the cath lab  B) Immediate transfer to the coronary care unit and schedule coronary angiography within 48 hours C) Immediate transfer to the coronary care unit and schedule coronary angiography within 24 hours | A | If B or C is selected:  The patient yells at you: “Doctor, I’m telling you I’m in pain!” His relatives rush in obvious distress and shout: “Can’t you see he’s in pain? Are you just going to sit there? Do something!” The attending physician recognizes the situation, calms everyone down, and asks you to assist with the catheterization as the patient is prepped for the catheterization lab. |
| Question 1 visual material:   | | |
| **2.** What medication will you administer to the patient?  A) Aspirin 325 mg and t-PA B) Aspirin 325 mg and unfractionated heparin (weight-adjusted) | B | If A is selected:  Shortly after administration and just before transfer to the cath lab, the patient develops a nosebleed: “Doctor, my nose is bleeding! What’s going on? What did you do to me?” You transfer him to the coronary unit and consult ENT to pack the bleeding site. Once stable, he is transported to the cath lab, now covered with weight-adjusted unfractionated heparin. |
| **3.** During transfer to the cath lab, the following rhythm appears on the monitor. How do you proceed?  A) Administer atropine B) Administer adrenaline C) Administer amiodarone | A | If B or C is selected:  The condition remains unchanged and the BP rises. The nurse beside you says: “Doctor, this is a pause! You need to give atropine.” |
| Question 3 visual material:   | | |
| **4.** Five minutes later, a similar rhythm appears on the monitor. How do you proceed?  A) Atropine continuous infusion B) Temporary pacemaker insertion C) Synchronized shock after anesthesia D) Unsynchronized shock after anesthesia | B | If A is selected:  Nurse: “Continuous atropine? It looks like you are new here. He needs a temporary pacemaker, doctor!” If C is selected: Anesthesiologists: “You consulted us for this? You shock tachycardias, not pauses! He needs a pacemaker.” If D is selected: The monitor shows chaotic waves (VF). The patient collapses immediately, no pulse, no breathing. You initiate CPR, but the patient never regains circulation.  (This represents the first of four potentially fatal errors in the scenario. ☠️) |
| **5.** During the beginning of the coronary angiography, the patient shows BP 60/40 mmHg. What do you administer?  A) Immediate norepinephrine B) Immediate dobutamine C) Immediate digoxin (weight-based) | A | If B or C is selected:  No clinical change. The attending cardiologist: “What are you doing? What did you give? Give NORA! The pressure has dropped!” |
| **6.** During the angiographic evaluation, the right coronary system shows no significant stenosis. The left coronary system is shown. What is your next step?  A) Angioplasty of LAD B) Angioplasty of LAD and LCx C) Multivessel coronary artery disease – urgent surgical consult D) No intervention – MINOCA | A | If B is selected:  Technician: “Doctor, check the LCx again. I don’t think there’s a significant lesion.” If C or D is selected: The patient returns to the unit and says: “Doctor, I’m still in pain. Weren’t you supposed to open the vessel? I’m feeling even worse now.” He is urgently returned to the cath lab for angioplasty of the culprit LAD lesion. |
| Question 6 visual material:   | | |
| **7.** Before proceeding with angioplasty, how do you modify the patient’s medication?  A) Add ticagrelor B) Add tirofiban C) Add t-PA | A | If B or C is selected:  Before angioplasty, the patient develops a nosebleed. He is transferred to the coronary unit, ENT is consulted, and after stabilization, he returns to the cath lab. |
| 8. After placing the stent, the angiographic image is shown. What is your next step?  A) Successful angioplasty, transfer to coronary unit B) LAD perforation with hemopericardium – pericardiocentesis and covered stent C) No-reflow phenomenon – administer intracoronary fluids and nitrates | C | If A is selected:  In the unit, the patient says: “Doctor, I’m still in pain. Didn’t you fix it?” He returns to the lab where no-reflow is diagnosed and treated. If B is selected: While attempting the pericardiocentesis, you perforate the cardiac wall. The patient immediately collapses and dies despite CPR. (This represents the second of four potentially fatal errors in the scenario. ☠️) |
| Question 8 visual material:   | | |
| **9.** Shortly after the administration, the monitor shows the rhythm below. How do you proceed?  A) No intervention, monitor the patient B) Synchronized shock C) Amiodarone | A | If B is selected:  Anesthesiologists irritated: “This is reperfusion rhythm! It’s because the vessel reopened. Usually nothing needs to be done in this case!” If C is selected: HR drops but the rhythm remains unchanged. The attending: “Why did you give that? This is reperfusion rhythm! You should have left it as it was. Now you have to watch for bradycardia!” |
| Question 9 visual material:   | | |
| 10. The patient, now in the coronary unit, develops dyspnea. SpO₂ = 83% on nasal cannula 4 L/min, BP: 100/60. Besides increasing oxygen delivery, how do you proceed?  A) Nitrates and furosemide B) Salbutamol/ipratropium and furosemide C) Methylprednisolone and furosemide D) Immediate dialysis E) Furosemide | E | If A or D is selected:  Nurse: “You want to remove the water from his lungs, not drop his pressure! Furosemide is enough, doctor!” If B or C is selected: SpO₂ worsens. Attending: “He’s congested from pulmonary edema, not asthmatic! Furosemide is enough.” |
| Question 10 visual material:   | | |
| **11.** Despite furosemide, the patient remains anuric. How do you proceed?  A) Preserved EF – no further action needed B) Low EF – start dobutamine C) Mechanical complication suspected – back to cath lab | B | If A is selected:  Patient remains symptomatic: “Doctor, I can’t breathe!” Attending: “Give dobutamine! He’s symptomatic.”  If C is selected: Cath lab shows no complication. Attending: “Give dobutamine! He’s symptomatic.” |
| Question 11 visual material:   | | |
| **12.** Two hours later on the monitor, you see the following rhythm. Patient’s BP is 105/65 mmHg. How do you proceed?  A) Amiodarone B) Flecainide C) Adrenaline | A | If B is selected:  No effect. Nurse: “That’s VT. Give amiodarone!” If C is selected: VF appears. Patient collapses – CPR starts but is unsuccessful. (This represents the third of four potentially fatal errors in the scenario. ☠️) |
| Question 12 visual material:   | | |
| **13.** Before administering amiodarone, the patient becomes altered. Rhythm unchanged, BP 60/35. How do you proceed?  A) Adrenaline B) Synchronized shock C) Unsynchronized shock | B | If A is selected:  VF appears. CPR started. Patient revived. If C is selected: VF appears. Patient collapses – CPR starts but is unsuccessful. (This represents the fourth of four potentially fatal errors in the scenario. ☠️) |
| **14.** Patient returns to sinus rhythm. How do you proceed?  A) Plan immediate ICD implantation B) Plan VT ablation C) Repeat angiography to check stent patency D) No intervention needed | D | If A, B, or C is selected:  VT within 48 hours of MI has no prognostic value (2023 ESC ACS Guidelines). No intervention needed at this time. |
| **15.** The patient remains hemodynamically stable for 72 hours after discontinuation of intravenous medications. The ejection fraction is estimated at 30%. Apart from DAPT and lipid-lowering therapy, what is the appropriate discharge treatment?  A) β-blocker, ACE inhibitor, amlodipine, SGLT2 inhibitor B) β-blocker, ARB, amlodipine, SGLT2 inhibitor C) β-blocker, ARB, ACE inhibitor, MRA, SGLT2 inhibitor D) β-blocker, ACE inhibitor, MRA, SGLT2 inhibitor | D | If A, B, or C is selected:  The four cornerstone medical therapies proven to improve survival in HFrEF are: a β-blocker, an ACE inhibitor or ARB, a mineralocorticoid receptor antagonist (MRA), and an SGLT2 inhibitor. |
| **16**. The patient has been discharged and, after 3 months of optimal, maximally tolerated medical therapy, returns for a scheduled follow-up. He is currently classified as NYHA functional class II. Echocardiography reveals an ejection fraction (EF) of 30%. The ECG obtained during this visit is shown below. What is the next appropriate step in management?  A) Implantation of an ICD for primary prevention should be scheduled  B) Implantation of a biventricular pacemaker (CRT-P) should be scheduled C) Implantation of a biventricular pacemaker-defibrillator (CRT-D) should be scheduled | A | If B or C is selected:  “The patient has ischemic HFrEF, more than 3 months post-MI, and is on optimal maximally tolerated GDMT. According to the 2022 ESC guidelines on ventricular arrhythmia management and sudden cardiac death prevention, an ICD should be implanted for primary prevention of ventricular arrhythmias. In this case, CRT is not indicated, as QRS duration is <130 ms, based on the 2021 ESC guidelines on pacing and CRT.” |
| Question 16 visual material:   | | |
| **End of scenario.** | | |
| Abbreviations: ACE inhibitor (Angiotensin-Converting Enzyme inhibitor), ACS (Acute Coronary Syndrome), ARB (Angiotensin II Receptor Blocker), BP (Blood Pressure), Cath lab (Catheterization Laboratory), CD-VP (Clinician-developed virtual patient), CPR (Cardiopulmonary Resuscitation), CRT (Cardiac Resynchronization Therapy), CRT-D (Cardiac Resynchronization Therapy with Defibrillator), CRT-P (Cardiac Resynchronization Therapy with Pacemaker), DAPT (Dual Antiplatelet Therapy), EF (Ejection Fraction), ENT (Ear, Nose, and Throat), ECG (Electrocardiogram), ESC (European Society of Cardiology), GDMT (Guideline-Directed Medical Therapy), HFrEF (Heart Failure with Reduced Ejection Fraction), HR (Heart Rate), ICD (Implantable Cardioverter-Defibrillator), IV (Intravenous), LAD (Left Anterior Descending artery), LCx (Left Circumflex artery), MINOCA (Myocardial Infarction with Non-Obstructive Coronary Arteries), MI (Myocardial Infarction), MRA (Mineralocorticoid Receptor Antagonist), NYHA (New York Heart Association), SGLT2 inhibitor (Sodium-Glucose Cotransporter 2 Inhibitor), SpO₂ (Peripheral Capillary Oxygen Saturation), t-PA (Tissue Plasminogen Activator), VF (Ventricular Fibrillation), VT (Ventricular Tachycardia). | | |

# Section B. Full AI-Generated, Expert-Reviewed Virtual Patient Scenario (GPT-VP)

| **GPT-VP: Question No.** | **Correct response** | **Incorrect Responses & Feedback** |
| --- | --- | --- |
| 1. You are a Cardiology resident in the Emergency Department. A 58-year-old man with a history of arterial hypertension and hyperlipidemia presents with sudden-onset chest tightness while walking to his car. The pain radiates to the jaw, started 35 minutes ago, and is accompanied by sweating and nausea.  Vital signs: heart rate 94/min, blood pressure 138/84 mmHg, respiratory rate 18/min, oxygen saturation (SpO₂) 98% on room air.  Electrocardiogram (ECG): ST-segment elevation in leads II, III, and aVF.  What is the most appropriate next step in management? A) Administer nitroglycerin and reassess in 15 minutes B) Start dual antiplatelet therapy and prepare for PCI C) Perform echocardiography and order serial troponins | B | If A is selected: Nurse: “The pain is getting worse and we still have not activated the catheterization laboratory.”  If C is selected: Attending physician: “Troponins may confirm the infarction, but PCI should not be delayed.”  Rationale: This is an inferior ST-elevation myocardial infarction (STEMI); immediate dual antiplatelet therapy and primary percutaneous coronary intervention (pPCI) are indicated. |
| Question 1 visual material:  | | |
| 2. During transfer to the catheterization laboratory, which antiplatelet agent should be administered together with aspirin? A) Prasugrel (P2Y12 inhibitor) B) Ticagrelor (P2Y12 inhibitor) C) Clopidogrel (P2Y12 inhibitor) | B | If A is selected: Attending physician: “We do not yet know whether he will need surgery. Prasugrel may not be safe.”  If C is selected: Attending physician: “Clopidogrel is an acceptable option, but ticagrelor is usually preferred unless there is a contraindication.”  Rationale: Ticagrelor was selected in this scenario because it provides rapid and potent platelet inhibition and can be administered before PCI when prasugrel suitability is uncertain. Prasugrel should be avoided in patients with prior stroke/TIA and used only after contraindications and procedural suitability have been considered. Clopidogrel is generally reserved for cases in which ticagrelor or prasugrel are contraindicated or unavailable. |
| Question 2 visual material:   | | |
| 3. During catheterization, the right coronary artery (RCA) is found to be 99% occluded. The patient develops hypotension (BP 82/48 mmHg) and bradycardia. What is your first step? A) Administer intravenous atropine B) Dopamine infusion C) Temporary transvenous pacing | A | If B is selected: Attending physician: “Let us try atropine first before starting vasopressors — the heart rate is the main problem.”  If C is selected: Nurse: “Should we not try pharmacological treatment first?”  Rationale: Symptomatic bradycardia with hypotension during inferior STEMI is usually due to increased parasympathetic tone; atropine is the first-line treatment. |
| Question 3 visual material:  | | |
| 4. After stent placement, the patient develops narrow-QRS tachycardia with a regular rhythm (heart rate 160/min). He is hemodynamically stable. What is the next step? A) Administer adenosine B) Synchronized cardioversion C) Administer intravenous amiodarone | A | If B is selected: Anesthesiologist: “He is alert and hemodynamically stable — cardioversion is not needed.”  If C is selected: Team member: “Adenosine acts faster — let us try that first.”  Rationale: Stable supraventricular tachycardia is treated first with adenosine for diagnostic and therapeutic purposes, including rhythm termination. |
| Question 4 visual material:  | | |
| 5. A little later, the heart rate (HR) falls to 42 bpm, but arterial pressure remains stable. What do you do? A) Monitor with continuous recording B) Start dopamine infusion C) Insert a pacemaker | A | If B is selected: Attending physician: “Vasoactive drugs are not needed unless hypotension develops.”  If C is selected: Nurse: “His blood pressure remains normal — there is no indication for pacing yet.”  Rationale: Asymptomatic bradycardia after reperfusion can be monitored, provided there is hemodynamic stability. |
| Question 5 visual material:  | | |
| 6. On day 3, the left ventricular ejection fraction (LVEF) is 30%. Which pharmacological treatment should be started at discharge? A) Beta-blocker, ACE inhibitor, mineralocorticoid receptor antagonist (MRA), and SGLT2 inhibitor B) Beta-blocker and loop diuretic C) Beta-blocker, angiotensin receptor blocker (ARB), and digoxin | A | If B is selected: Attending physician: “We need to start guideline-directed therapy — not only symptomatic treatment.”  If C is selected: Attending physician: “Digoxin is not a first-line drug here — start the core heart-failure medications.”  Rationale: The combination of a beta-blocker, ACE inhibitor, MRA, and SGLT2 inhibitor reduces mortality and rehospitalizations in patients with heart failure with reduced ejection fraction (HFrEF) after myocardial infarction. |
| Question 6 visual material:   | | |
| 7. Two days later, serum creatinine rises to 2.3 mg/dL (from a baseline value of 1.1 mg/dL), and urine output decreases. What is the appropriate management? A) Discontinue the ACE inhibitor B) Administer intravenous fluids C) Start renal replacement therapy (dialysis) | A | If B is selected: Nurse: “He has fluid retention — intravenous fluids will make him worse.”  If C is selected: Nephrologist: “There is no indication for dialysis yet — manage him conservatively for now.”  Rationale: ACE inhibitors can contribute to renal dysfunction, particularly when renal perfusion is compromised. A clinically significant creatinine rise with oliguria warrants withholding the ACE inhibitor, assessing volume status, avoiding unnecessary intravenous fluids if the patient is congested, and reassessing renal function. Renal replacement therapy is reserved for standard indications. |
| 8. Four days after the infarction, the patient develops acute chest pain that worsens with inspiration. The ECG shows diffuse ST-segment elevation and PR-segment depression. What is the most likely diagnosis and treatment? A) Post-myocardial infarction pericarditis – high-dose aspirin/NSAID therapy B) Recurrent STEMI – immediate coronary angiography C) Pulmonary embolism – anticoagulation | A | If B is selected: Fellow: “Diffuse ST elevations and PR depression — this is pericarditis, not STEMI.”  If C is selected: Attending physician: “There are no clinical signs of pulmonary embolism — the ECG and the pain support pericarditis.”  Rationale: Classic signs of pericarditis include chest pain that worsens with inspiration and diffuse ST-segment elevations with PR-segment depression on ECG. High-dose aspirin or NSAID therapy is first-line anti-inflammatory treatment after careful clinical assessment; aspirin is often preferred in the post-MI setting, and colchicine may be considered as adjunctive therapy depending on context. |
| Question 8 visual material:  | | |
| 9. During follow-up, the ECG shows a QTc interval of 530 ms. What is the next step? A) Check electrolytes B) Start amiodarone C) Schedule pacemaker implantation | A | If B or C is selected: The team redirects management toward identifying reversible causes rather than adding QT-prolonging therapy or device treatment.  Rationale: The first-line approach is correction of reversible causes, such as electrolyte abnormalities and discontinuation of medications that prolong the QT interval. |
| 10. Three months later, the ejection fraction (EF) remains 30%. The QRS duration is 160 ms with left bundle branch block (LBBB). What is the next step? A) Referral for implantation of a biventricular pacemaker-defibrillator (CRT-D) B) Implantation of an ICD only C) Repeat coronary angiography | A | If B is selected: Attending physician: “He also meets criteria for CRT — not only for ICD.”  If C is selected: Interventional cardiologist: “He has already undergone complete revascularization — we do not suspect ischemia.”  Rationale: According to ESC guidelines, this patient with HFrEF (LVEF 30%), QRS >130 ms, and LBBB is suitable for CRT-D. |
| Question 10 visual material:   | | |
| 11. At 3 months, the patient remains mildly symptomatic. He is receiving full pharmacological treatment. Heart rate (HR) is 90 bpm. What is the next step? A) Add ivabradine B) Switch to digoxin C) Continue the current treatment without changes | A | If B is selected: Attending physician: “Digoxin is not used routinely in HFrEF unless atrial fibrillation is present.”  If C is selected: Attending physician: “He is NYHA class II — we should optimize his therapy.”  Rationale: Ivabradine is indicated in patients with HFrEF and HR >70 bpm despite the maximum tolerated dose of a beta-blocker, with the aim of reducing symptoms and hospitalizations. |
| Question 11 visual material:   | | |
| 12. The patient neglects systematic adherence to his medication and returns to his previous habits. Two months later, he develops chest pain again. Because of its similarity to the previous episode, he promptly presents to the Cardiology Emergency Department, where the ECG shows ST-segment depression in the lateral leads and troponins are positive. What is the most likely diagnosis? A) Non-ST-elevation myocardial infarction (NSTEMI) B) ST-elevation myocardial infarction (STEMI) C) Unstable angina (UA) | A | If B is selected: Fellow: “There is no ST elevation — therefore this is not STEMI.”  If C is selected: Attending physician: “The positive troponin excludes unstable angina.”  Rationale: Ischemic ECG changes together with elevated troponins confirm the diagnosis of NSTEMI. |
| 13. Which anticoagulant is preferred in the management of NSTEMI? A) Enoxaparin B) Warfarin C) Alteplase | A | If B is selected: Attending physician: “Warfarin is not used in acute coronary syndromes — do not confuse this with atrial fibrillation.”  If C is selected: Nurse: “That is a thrombolytic — it is not indicated for NSTEMI.”  Rationale: Low-molecular-weight heparin (LMWH), such as enoxaparin, is the standard treatment in patients with NSTEMI. |
| Question 13 visual material:   | | |
| 14. During the stay in the coronary care unit, the patient shows the following findings on chest radiography. Arterial pressure is 160/90 mmHg. What do you do? A) Administer intravenous furosemide plus nitroglycerin B) Start dialysis C) Administer inhaled salbutamol | A | If B is selected: Nephrologist: “He does not have diuretic resistance — treat the congestion.”  If C is selected: Fellow: “This is not bronchospasm — it is cardiogenic pulmonary edema.”  Rationale: Hypertensive pulmonary edema is treated with diuretics and afterload reduction using vasodilators. |
| Question 14 visual material:  | | |
| 15. The current acute coronary syndrome was managed successfully. After the coronary angiography result and discharge, the patient appears to understand the need to control risk factors. What is the LDL-cholesterol (LDL-C) target value for this patient? A) <55 mg/dL B) <100 mg/dL C) <40 mg/dL | C | If A or B is selected: The target is more stringent because this is a second acute coronary syndrome within 2 years.  Rationale: According to the latest ESC guidelines, because there was a second ACS episode within 2 years, the LDL-C target is <40 mg/dL, together with a ≥50% reduction from baseline. |
| Question 15 visual material:   | | |
| 16. The patient asks whether there is a medication he will most likely need to take for the rest of his life. What do you answer? A) Aspirin B) Ticagrelor C) Loop diuretic | A | If B is selected: Ticagrelor is usually discontinued after 12 months, individualized according to ischemic and bleeding risk.  If C is selected: Loop diuretics have a symptomatic role, primarily for decongestion in heart failure or edema.  Rationale: Aspirin is continued lifelong after ACS/PCI unless contraindicated. The second antiplatelet agent, such as ticagrelor, is usually stopped after 12 months, with individualized adjustment according to ischemic and bleeding risk. Diuretics have a symptomatic role for relief of congestion.  Congratulations, you treated the patient and helped him change his way of life! |
| Question 16 visual material:   | | |
| **End of scenario.** | | |
| Abbreviations: ACE inhibitor (Angiotensin-Converting Enzyme inhibitor), ACS (Acute Coronary Syndrome), ARB (Angiotensin II Receptor Blocker), BP (Blood Pressure), bpm (beats per minute), CRT (Cardiac Resynchronization Therapy), CRT-D (Cardiac Resynchronization Therapy with Defibrillator), DAPT (Dual Antiplatelet Therapy), ECG (Electrocardiogram), EF (Ejection Fraction), ESC (European Society of Cardiology), HFrEF (Heart Failure with Reduced Ejection Fraction), HR (Heart Rate), ICD (Implantable Cardioverter-Defibrillator), IV (Intravenous), LBBB (Left Bundle Branch Block), LDL-C (Low-Density Lipoprotein Cholesterol), LMWH (Low-Molecular-Weight Heparin), LVEF (Left Ventricular Ejection Fraction), MRA (Mineralocorticoid Receptor Antagonist), NSAIDs (Nonsteroidal Anti-Inflammatory Drugs), NSTEMI (Non-ST-Elevation Myocardial Infarction), NYHA (New York Heart Association), PCI (Percutaneous Coronary Intervention), pPCI (Primary Percutaneous Coronary Intervention), P2Y12i (P2Y12 inhibitor), QRS (Q, R, and S waves of the electrocardiogram), QTc (corrected QT interval), RCA (Right Coronary Artery), SGLT2 inhibitor (Sodium-Glucose Cotransporter 2 Inhibitor), SpO₂ (Peripheral Capillary Oxygen Saturation), STEMI (ST-Elevation Myocardial Infarction), SVT (Supraventricular Tachycardia), UA (Unstable Angina). | | |

**Note.** CD-VP = clinician-developed virtual patient; GPT-VP = ChatGPT-generated virtual patient; MobiViP = mobile virtual patients app; VP = virtual patient.
