# Supplementary material for: Perceived Ratings of AI-Generated and Expert-Reviewed vs Clinician-Developed Virtual Cardiology Patient Scenarios Among Medical Students: Randomized Controlled Study
Source: JMIR Med Educ. 2026 Oct 28;12:e100839. doi: 10.2196/100839 (PMC13755779; doi:10.2196/100839)
Supplement: Supplementary file 4 [file mededu_v12i1e100839_app4.docx]

**Graphical abstract**

**Note:** Values shown for the four domains are mean subscale scores on a 5-point Likert scale. *P* values refer to between-group comparisons using Mann-Whitney *U* tests. CD-VP: clinician-developed virtual patient; ECG: electrocardiogram; GPT-VP: ChatGPT-generated virtual patient; MobiViP: mobile virtual patients app; VP: virtual patient.
